# An overview of the Mediterranean cave-dwelling horny sponges (Porifera, Demospongiae)

**DOI:** 10.3897/zookeys.281.4171

**Published:** 2013-03-28

**Authors:** Renata Manconi, Barbara Cadeddu, Fabio Ledda, Roberto Pronzato

**Affiliations:** 1 Università di Sassari, Dipartimento di Scienze della Natura e del Territorio, Italy; 2 Università di Genova, Dipartimento di Scienze della Terra, dell’Ambiente e della Vita, Italy

**Keywords:** Biodiversity, marine caves, taxonomy, checklist, diagnostic keys, Dendroceratida, Dictyoceratida, Halisarcida, Verongida

## Abstract

The present synthesis focuses on the so called ‘horny sponges’ recorded from marine caves of the Mediterranean Sea. The main aim is to provide a list of all recorded species, diagnostic keys to their identification up to family and genus level, and exhaustive, formally uniform descriptions at the species level contributing to sharing of information on the faunistics and taxonomy of Mediterranean cave-dwelling species, including habitat preferences. The majority of species was recorded in 105 Mediterranean marine caves hosting four orders of horny sponges belonging to 9 families, 19 genera and 40 species. Species endemic to the Mediterranean Sea harboured in marine caves are 14 with an endemicity value of 35%. For each species morphological descriptions are supported by illustrations both original and from the literature, including the diagnostic traits of the skeleton by light and scanning electron microscopy giving further characterization at the specific level. A detailed map together with a list of all caves harbouring horny sponges is also provided with geographic coordinates.

## Introduction

The Mediterranean area represents a hot spot of biodiversity and needs more and deeper studies together with urgent conservation plans on its marine biocoenosis and ecosystems. Among dominant benthic taxa Mediterranean sponge species number over 600 with a high endemicityvalue (*ca*. 40%) (Pansini and Longo 2003, 2008; Pronzato 2003; Pansini et al. 2011). The horny sponge fauna also is characterized by high levels of endemism (18 species=31.6% endemicity) from all Mediterranean biotopes (Pansini 1992; Pansini and Longo 2003, 2008; Pronzato 2003; Voultsiadou 2005; Pronzato and Manconi 2011). Although the last synthesis by Van Soest et al. (2012a) reports 654 species, 203 genera, and 86 families of Porifera, the real species richness of the Mediterranean Sea is, apparently, highly over- or under-estimated.

As far as vulnerable biotopes such as marine caves are concerned, data on sponges are scattered widely in the literature and several records are published in not easily accessible regional journals or books. After the pioneering work of Michele Sarà, who collected cave-dwelling sponges by snorkelling in semi-submerged (mid-littoral) caves (Sarà 1958), sampling methods by SCUBA diving highly improved data on biodiversity also from submerged caves (Riedl 1966; Rützler 1966). Results on cave-dwelling sponges highlighted the fact that the taxon Porifera is dominant in these cryptic Mediterranean biotopes, performing a key role in the benthic community structure of caves.

**Figure 1.**
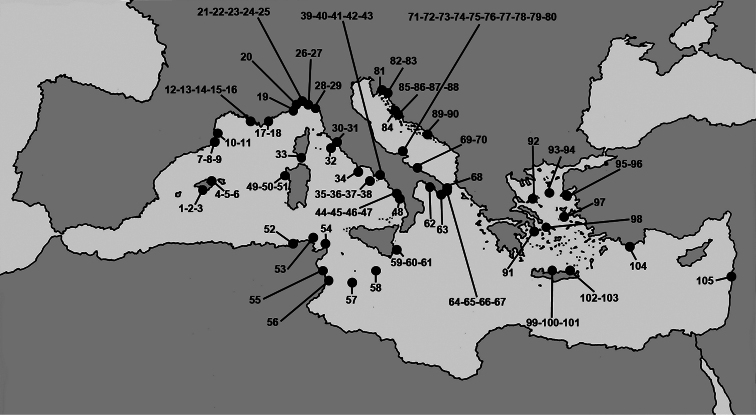
Mediterranean marine caves. Numbers refer to the caves from which horny sponge species are reported.

The present paper reports all known records of the horny sponges (Orders Dendroceratida, Dictyoceratida, Halisarcida,Verongida) from a wide array of marine caves in the entire Mediterranean Sea with a checklist and diagnostic keys to benefit an online open-access supporting global sharing of information on faunistics and taxonomy (Fig. 1; Tables 1, 2). Exhaustive and formally uniform morphological descriptions of species are provided although some were previously reported in part by Pronzato and Manconi (2011) in a rather regional and not widely accessible data source.

**Table 1.** Marine caves harbouring horny sponges in sub-basins of the Mediterranean Sea with geographic coordinates. New records in recently investigated karstic caves are indicated by asterisks. Cave numbers refer to the map in Fig. 1.

	**Balearic Sea**		
1	Calamars Cave	39°07'N, 02°55'E
2	Blue Cave	39°07'N, 02°55'E
3	Blava Cave	39°09'N, 02°55'E
4	La Catedral Cave	39°44'N, 03°27'E
5	J 1 Cave	39°44'N, 03°27'E
6	J 2 Cave	39°44'N, 03°27'E
7	Meda Petita Cave	42°02'N, 03°13'E
8	Misidacis Cave	42°02'N, 03°13'E
9	Petita de la Vaca Cave	42°03'N, 03°12'E
	**Gulf of Lions**		
10	Troc Cave	42°28'N, 03°08'E
11	Béar Cave	42°30'N, 03°08'E
12	Niolon Cave	43°20'N, 05°15'E
13	Endoume Cave	43°16'N, 05°21'E
14	Corail Cave	43°12'N, 05°19'E
15	Figuier Cave	43°12'N, 05°26'E
16	Trèmies Cave	43°12'N, 05°31'E
17	Bagaud caves	43°00'N, 06°23'E
18	Pointe des Carrieres Cave	42°59'N, 06°12'E
	**Ligurian Sea**		
19	Gallinara Island Cave	44°01'N, 08°13'E
20	Bergeggi Island Cave	44°13'N, 08°26'E
21	Punta Carega Cave	44°18'N, 09°12'E
22	Western-Zoagli Cave	44°20'N, 09°16'E
23	Zoagli-Chiavari Cave	44°19'N, 09°17'E
24	Piccola Zoagli-Chiavari Cave	44°19'N, 09°17'E
25	Punta Manara Cave	44°15'N, 09°24'E
26	Western-Bonassola Cave	44°11'N, 09°35'E
27	Eastern-Bonassola Cave	44°11'N, 09°35'E
28	Tinetto Cave	44°01'N, 09°51'E
29	Lerici Cave	44°04'N, 09°55'E
	**Central Tyrrhenian Sea**		
30	Isolotto Cave	42°23'N, 11°13'E
31	Azzurra Cave-Porto Ercole	42°22'N, 11°12'E
32	Giannutri Cave	42°15'N, 11°06'E
33	Bonifacio Cave	41°23'N, 09°09'E
34	Ponza Cave	40°53'N, 12°57'E
35	Monte Vico Cave	40°45'N, 13°53'E
36	Lacco Ameno caves	40°45'N, 13°53'E
37	Secca Formiche-Vivara Cave	40°43'N, 13°58'E
38	Mago Cave	40°42'N, 13°58'E
39	Misteri Cave	40°47'N, 14°10'E
40	Gaiola Cave	40°47'N, 14°10'E
41	Scraio-Vico Equense Cave	40°39'N, 14°25'E
42	Tuffo Tuffo Cave	40°37'N, 14°21'E
43	Mitigliano Cave	40°35'N, 14°19'E
	**Southern Tyrrhenian Sea**		
44	Azzurra Cave-Policastro	39°59'N, 15°22'E
45	Infreschi Cave	39°59'N, 15°22'E
46	Molare Cave	40°03'N, 15°29'E
47	Maratea Cave	40°00'N, 15°43'E
48	Leone Cave	39°52'N, 15°46'E
	**Sardinian Sea**		
49	Galatea Cave ^*^	40°34'N, 08°13'E
50	Falco Cave ^*^	40°34'N, 08°13'E
51	Bisbe Cave ^*^	40°34'N, 08°12'E
	**Sicily Channel**		
52	Tabarka Tunnel	36°58'N, 08°45'E
53	Cani Islands Tunnel	37°21'N, 10°07'E
54	Zembra caves	37°07'N, 10°48'E
55	Monastir caves	35°47'N, 10°49'E
56	Salakta caves	35°23'N, 11°03'E
57	Taccio Vecchio I Cave ^*^	35°31'N, 12°35'E
58	Gozo Cave	36°02'N, 14°15'E
	**Ionian Sea**		
59	Mazzere Cave ^*^	37°00'N, 15°18'E
60	Gamberi Cave ^*^	37°00'N, 15°19'E
61	Gymnasium Cave ^*^	37°00'N, 15°18'E
62	Porto Cesareo Cave	40°15'N, 17°54'E
63	Leuca caves	39°47'N, 18°21'E
64	Principessa Cave	39°48'N, 18°22'E
65	Marinella Cave	39°49'N, 18°23'E
66	Piccola del Ciolo Cave	39°50'N, 18°23'E
67	Sifone Cave	39°52'N, 18°23'E
68	Castro Marina Cave	39°59'N, 18°25'E
	**Southern Adriatic Sea**		
69	Torre Incine Cave	40°59'N, 17°16'E
70	Regina Cave	41°05'N, 16°59'E
71	Rondinelle Cave	42°06'N, 15°28'E
72	Viole Cave	42°06'N, 15°29'E
73	Bue Marino Cave	42°06'N, 15°29'E
74	Pecore Cave	42°06'N, 15°29'E
75	Pagliai Cave	42°07'N, 15°29'E
76	Arenile Cave	42°07'N, 15°29'E
77	Coccodrillo Cave	42°07'N, 15°29'E
78	Cala Tonda Cave	42°07'N, 15°29'E
79	Cala Spido Cave	42°07'N, 15°30'E
80	Cala Sorrentino Cave	42°08'N, 15°30'E
	**Northern Adriatic Sea**		
81	Columbera Cave	45°10'N, 14°14'E
83	Cave near Vrbnik	45°04'N, 14°40'E
83	Strazica Cave	44°56'N, 14°46'E
84	Katedrala Cave	44°18'N, 14°38'E
85	Y Cave	44°03'N, 14°59'E
86	Golubinka Cave	44°03'N, 14°59'E
87	Submarine Passage Cave	44°03'N, 14°59'E
88	Garmenjak Cave-Veli Island	43°52'N, 15°11'E
89	Island Bratin Cave	42°44'N, 16°47'E
90	Medvjeđa Cave-Lastovo Isl.	42°45'N, 16°52'E
	**Aegean Sea**		
91	Vouliagmeni Cave	37°47'N, 23°47'E
92	Youra Island Cave	39°23'N, 24°09'E
93	Ftelio Cave	39°30'N, 24°58'E
94	Trypia Spilia Cave	39°32'N, 24°58'E
95	Farà Cave	38°58'N, 26°28'E
96	Agios Vasilios Cave	38°58'N, 26°32'E
97	Chios (station 213)	38°11'N, 26°16'E
98	Andros Cave	37°48'N, 24°58'E
99	Stravos Cave	35°25'N, 24°58'E
100	Alykes Cave	35°25'N, 24°59'E
101	Madhes Cave	35°24'N, 25°02'E
102	Agio Nicolaos cave	35°11'N, 25°43'E
103	Gournia Cave	35°07'N, 25°46'E
104	Kastelorizo (Megisti) Cave	36°02'N, 29°38'E
	**Levantine Basin**		
105	Raouché Cave	33°53'N, 35°28'E

**Table 2.** Checklist of Mediterranean cave-dwelling horny sponges. New records (18 species) in recently investigated karstic caves from Capo Caccia-Isola Piana MPA (Galatea, Falco, Bisbe), the Plemmirio MPA (Mazzere, Gamberi, Gymnasium), and the Pelagie MPA (Taccio Vecchio I, Lampedusa) are indicated by asterisks. Protected species of the protocol SPA/BIO are indicated by black spots.

**DENDROCERATIDA MINCHIN, 1900**
**DARWINELLIDAE MEREJKOWSKY, 1879**
***Aplysilla* Schulze, 1878**
*Aplysilla rosea* (Barrois, 1876) *
***Chelonaplysilla* de Laubenfels, 1948**
*Chelonaplysilla noevus* (Carter, 1876)
***Darwinella* Müller, 1865**
*Darwinella australiensis* Carter, 1885 *
*Darwinella* sp.
***Dendrilla* von Lendelfeld, 1883**
*Dendrilla* sp.
**DICTYODENDRILLIDAE BERGQUIST, 1980**
***Spongionella* Bowerbank, 1862**
*Spongionella gracilis* (Vosmaer, 1883)
*Spongionella pulchella* (Sowerby, 1804)
**DICTYOCERATIDA MINCHIN, 1900**
**DYSIDEIDAE GRAY, 1867**
***Dysidea* Johnston, 1842**
*Dysidea avara* (Schmidt, 1862) *
*Dysidea fragilis* (Montagu, 1818) *
*Dysidea incrustans* (Schmidt, 1862) *
*Dysidea tupha* (Martens, 1824)
*Dysidea* sp.
***Euryspongia* Row, 1911**
*Euryspongia raouchensis* Vacelet, Bitar, Carteron, Zibrowius & Perez, 2007
***Pleraplysilla* Topsent, 1905**
*Pleraplysilla minchini* Topsent, 1905
*Pleraplysilla spinifera* (Schulze, 1878) *
*Pleraplysilla* sp.
**IRCINIIDAE GRAY, 1867**
***Ircinia* Nardo, 1833**
*Ircinia dendroides* (Schmidt, 1862) *
*Ircinia oros* (Schmidt, 1864) *
*Ircinia paucifilamentosa* Vacelet, 1961
*Ircinia retidermata* Pulitzer-Finali & Pronzato, 1980
*Ircinia variabilis* (Schmidt, 1862) *
*Ircinia* sp.
***Sarcotragus* Schmidt, 1862**
*Sarcotragus fasciculatus* (Schmidt, 1862)
*Sarcotragus foetidus* (Schmidt, 1862) * •
*Sarcotragus pipetta* (Schmidt, 1868) •
*Sarcotragus spinosulus* (Schmidt, 1862)
*Sarcotragus* sp.
**SPONGIIDAE GRAY, 1867**
***Coscinoderma* Carter, 1883**
*Coscinoderma sporadense* Voultsiadou-Koukoura, van Soest & Koukouras, 1991
***Hippospongia* Schulze, 1879**
*Hippospongia communis* (Lamarck, 1813)
***Spongia* Linnaeus, 1759**
*Spongia lamella* (Schulze, 1879) * •
*Spongia nitens* (Schmidt, 1862) *
*Spongia officinalis* Linnaeus, 1759 * •
*Spongia virgultosa* (Schmidt, 1868) *
*Spongia zimocca* Schmidt, 1862 * •
*Spongia* sp.
**THORECTIDAE BERGQUIST, 1978**
***Cacospongia* Schmidt, 1862**
*Cacospongia mollior* Schmidt, 1862
*Cacospongia proficens* Pulitzer-Finali & Pronzato, 1980 *
*Cacospongia scalaris* Schmidt, 1862
***Fasciospongia* Burton, 1934**
*Fasciospongia cavernosa* (Schmidt, 1862) *
*Fasciospongia* sp.
***Hyrtios* Duchassaing & Michelotti, 1864**
*Hyrtios collectrix* (Schulze, 1879)
**HALISARCIDA BERGQUIST, 1996**
**HALISARCIDAE SCHMIDT, 1862**
***Halisarca* Johnston, 1842**
*Halisarca dujardini* Johnston, 1842
**VERONGIDA BERGQUIST, 1978**
**APLYSINIDAE CARTER, 1875**
***Aplysina* Nardo, 1834**
*Aplysina aerophoba* (Nardo, 1833) •
*Aplysina cavernicola* (Vacelet, 1959) •
*Aplysina* sp.
**IANTHELLIDAE HYATT, 1875**
***Hexadella* Topsent, 1896**
*Hexadella crypta* Reveillaud, Allewaert, Pérez, Vacelet, Banaigs & Vanreusel, 2012
*Hexadella pruvoti* Topsent, 1896
*Hexadella racovitzai* Topsent, 1896
*Hexadella topsenti* Reveillaud, Allewaert, Pérez, Vacelet, Banaigs & Vanreusel, 2012

## Taxonomy of “horny” sponges

Horny sponges, belonging to the class Demospongiae, are not a formal taxonomic group but in their evolutionary history they have shown a tendency to lose the trait typical of the class, namely the ability to produce a mineral siliceous skeleton. In the past, horny (= fibrous, *sensu* Bergquist, 1996) sponges were all included in the order Keratosa. The credit for this name is given by Grant (1861, p. 159), or Bowerbank (1862, p. 1118) as reported by de Laubenfels (1948).

Minchin (1900) split the Keratosa in Dendroceratida and Dictyoceratida. A further split into four orders occurred when Verongida and Halisarcida were erected in 1978 and 1996, respectively, under the authority of Bergquist. The current 4 orders include 11 families: Aplysinellidae Bergquist, 1980; Aplysinidae Carter, 1875; Darwinellidae Merejkowsky, 1879; Dictyodendrillidae Bergquist, 1980; Dysideidae Gray, 1867; Halisarcidae Schmidt, 1862; Ianthellidae Hyatt, 1875; Irciniidae Gray, 1867; Pseudoceratinidae Carter, 1885; Spongiidae Gray, 1867; Thorectidae Bergquist, 1978.

**Figure 2:**
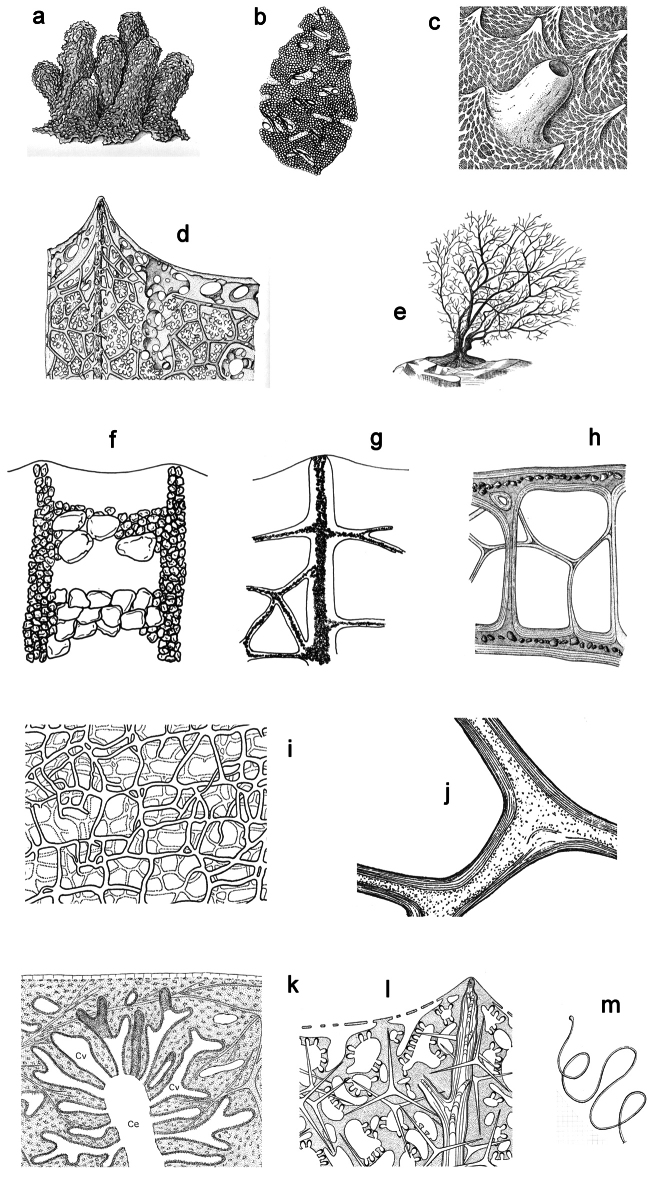
Horny sponge skeleton. All orders to which horny sponges belong share a wide array of growth form supported by skeletal architecture of spongin ranging from dendritic-arborescent to reticulate network, with fibres filled or not by mineral detritus **a** digitate growth form with conulose surface is a very common trait, but also massive or encrusting habits are displayed by a number of species **b** the sponge surface is, in several species, armed by granular mineral debris sometimes appearing as ornamentation; c) reticulate fibrose surface of an encrusting horny sponge species with the osculum surrounded by conules **d** vertical section of a conule supported by an ascending primary fibre, with mineral inclusions, connected with a network of thinner secondary fibres free of inclusions **e** the dendritic skeleton is sometimes ramified **f, g, h** differently cored primary and secondary fibres network **i** skeletal network composed only by secondary fibres free of inclusions **j** detail of the opaque fibrillar medulla coring the skeleton of some horny sponge species **k** the absence of an horny skeleton occur only in a few species **l** triradiate horny spicules free in the skeleton characterize a few sponge species **m** thin long filaments ending in a rounded button (knob) are an exclusive diagnostic trait of the family Irciniidae. Modified from several historical sources.

Three orders *viz*. Dendroceratida, Dictyoceratida, and Verongida, share the diagnostic traits of a ‘skeletal network exclusively of spongin fibres’ and the ‘absence of a mineral skeleton’ (Fig. 2). On the other hand the status of the fourth order Halisarcida, classically included among horny sponges, is always strongly debated for the trait ‘total absence of a fibrous skeleton’.

Systematics and phylogenetic relationships of horny sponges have only recently begun to be tested using current biochemical and molecular approaches, partly confirming the classical morphological classification scheme (Borchiellini et al. 2004; Lavrov et al. 2008; Erpenbeck et al. 2007, 2012). Molecular analyses showed that Dictyoceratida, Dendroceratida, Verongida, and Halisarcida are in fact closely related (Borchiellini et al. 2004; Lavrov et al. 2008).

The order Halisarcida was recently suggested to be moved to the order Chondrosida (Erpenbeck and Wörheide 2007; Ereskovsky et al. 2011). The phylogenetic tree based on molecular data (Ereskovsky et al. 2011, fig. 46, p. 26) shows *Halisarca* spp. close to *Chondrilla nucula* although this status is weakly supported by the relationship in the same tree of *Halisarca* spp. with *Ephydatia muelleri* (Suborder Spongillina) and *Aplysina fulva* (Order Verongida). As a consequence the entire phylogenetic tree must be considered with caution (see also Erpenbeck et al. 2012). We have given these results serious consideration but assume a conservative approach until better diagnostic molecular markers are available; therefore, we maintain the traditional taxonomic status of the order Halisarcida.

Basic references on “Keratosa” are few (von Lendenfeld 1889; de Laubenfels 1948; Bergquist 1980a, b, 1996; Cook and Bergquist 2002; Bergquist and Cook 2002a, b, c; Pronzato and Manconi 2011). After the last fundamental worldwide taxonomic revision (Hooper and Van Soest 2002), 56 genera of sponges with fibrous skeletons are considered valid, although the final number of species at the global level is still under discussion.

The discovery of new taxa showed a continuous and constant increase up to the present (see Pronzato 2003). First data on the Mediterranean Sea as the type locality of horny sponges are reported in the 13th edition of Systema Naturae (Linnaeus 1789). Starting from the description of *Spongia officinalis* L., 1759 a total of 20 authors are involved from 1759 to 2007 in the discovery of new horny sponge species with a maximum of 2-3 new species per decade. Out of that trend is the period 1862–1938, of intense inventory activity resulting in the discovery of a high number of new species and genera by Emile Topsent, Oscar Schmidt and Franz Eilhard Schulze.

In recent times only a few studies were published on horny sponge fauna mostly reporting on restricted geographic areas of the Mediterranean Sea (Vacelet 1959; Pronzato 1975; Pulitzer-Finali and Pronzato 1976, 1980; Rubió-Lois et al. 1981; Voultsiadou-Koukoura and Koukouras 1993; Uriz and Maldonado 2000; Pronzato et al. 2004; Pronzato and Manconi 2008, 2011).

## Materials and methods

Specimens were collected, by the authors and others, using SCUBA diving. Specimens were preserved in 95% ethanol, 4% formaldehyde or dried. For specimens registered in collections we use acronyms published in the Systema Porifera (Hooper and Van Soest 2002).

A detailed study of the external morphology was performed on growth form, surface traits e.g. dimensions and topographic distribution of conules, oscules, and inhalant apertures. For species identification, skeleton preparations for light microscopy (LM) were made by hand dissection under a stereomicroscope, which were dried and mounted in Canada balsam or similar media under a cover slip. Similar preparations for Scanning Electron Microscopy (SEM) were air dried and attached to a stub with drops of silver glue. Preparations were viewed, measured, and photographed to characterize diagnostic micro-traits.

Morphological descriptions of cave dwelling-species refer basically both to recent analyses of specimens in the authors’ collections, of type materials, and/or original and historical descriptions, also in those cases in which taxa were first reported from other seas.

The cave-dwelling horny sponges were critically reviewed for synonymies and based on recent trends in taxonomy following, in part, Systema Porifera (Hooper and Van Soest 2002), Fauna d’Italia (Pansini et al. 2011; Pronzato and Manconi 2011), and taxonomic databases such as the World Porifera Database (WPD) and WoRMS (Van Soest et al. 2012b; www.marinespecies.org/porifera). For more detailed synonymies and distribution patterns of some all species see Pronzato and Manconi (2011). Some divergent points of view of the authors on the taxonomic status of a few taxa with respect to the previous papers fonts are discussed in the text.

### Study area

All studied caves are submerged or semi-submerged and, in most cases, the entrances are no more than 20 m in depth.

According to the areas investigated in the past by cave sponge workers and following previous biogeographical analyses the Mediterranean Sea was divided into 14 areas (Table 1), namely the Alboran Sea, Balearic Sea, Sardinian Sea, Gulf of Lions, Ligurian Sea, Northern Tyrrhenian Sea, Central Tyrrhenian Sea, Southern Tyrrhenian Sea, Sicily Channel, Ionian Sea, Northern Adriatic Sea, Southern Adriatic Sea, Aegean Sea, and the Levantine Basin (Van Soest 1994; Pansini and Longo 2003, 2008; Xavier and Van Soest 2012; Cadeddu 2012; Gerovasileiou and Voultsiadou 2012). Not a single record of cave-dwelling horny sponges is reported for the Alboran Sea or the Northern Tyrrhenian Sea.

Additional data on new records (Fig. 1; Tables 1, 2) have been included in the historical dataset after recent investigations in some Italian Marine Protected Areas (MPA) of seven submerged caves of the Capo Caccia-Isola Piana MPA (n=3), the Plemmirio MPA (n=3), and the Pelagie MPA (n=1) (Manconi et al. 2011; Cadeddu 2012). These new records are indicated by asterisks in the text.

## Taxonomic accounts

We use the obsolete designation “horny sponges” *sensu* von Lendenfeld (1889) not acting as greenhorn taxonomists but for convenience, to avoid listing all four orders that once were included in one, Keratosa *sensu* de Laubenfels (1948) whenever referring to the group. Because of the trait “absence of mineral spicules in the skeleton”, the taxonomy of “horny sponges” is based on fewer characters than the other demosponges. In general, some valuable diagnostic traits for a correct identification are the spatial organization of spongin fibres and collagenous filaments in the skeleton, the homogeneous or laminate architecture of fibres, and the presence/absence of exogenous mineral inclusions within spongin (Fig. 2). In other cases supplementary characters include the shape and dimension of flagellate chambers, the richness of cellular types, and larval architecture. The morphological plasticity of sponges (see Gaino et al. 1995) is one of the key problems for a correct identification of taxa bearing exclusively a fibrous skeleton (Pronzato et al. 2003) with a few morphological traits sometimes constrained by the influence of environmental parameters. In any case, first-hand experience of many species, including live material, is important for the difficult task of horny sponge identification at the species level.

The following keys are useful aids for understanding cave-dwelling horny sponge diversity, even if they are necessarily imperfect due to the incongruence and uncertainties still present in the field. The diagnostic keys reach the family or genus level, whereas identification at the species level is based on detailed descriptions and illustrations provided here. In a few cases the species are known only from the original description and there are no subsequent findings, and so no images support the diagnoses. Moreover the validity of some taxa is strongly under debate, in-depth revisions are needed and the possibility of synonymies is real. The present overview is systematically conservative and aims at facilitating the identification of Mediterranean cave-dwelling horny sponges.

**Order Dendroceratida Minchin, 1900**

**Diagnosis** (emended after Bergquist and Cook 2002a). Demospongiae with skeleton exclusively composed by horny fibres arising from a spongin basal plate. In one genus free fibrous spicules in the choanosome. No endogenous mineral elements in the skeleton. Fibres dendritically arranged as small, adjacent, ascending fibres, sometimes anastomosing. In a few genera a fibrous network characterises the skeleton (this diverging trait is problematic for the homogeneity of the taxon). Choanocyte chambers either diplodal (small, spherical) or eurypylous. Mode of reproduction viviparous. Larvae large, brooded parenchymellae with a posterior clump of long cilia.

**Order Dictyoceratida Minchin, 1900**

**Diagnosis **(emended after Cook and Bergquist 2002). Demospongiae with skeleton of horny fibres anastomosing and, often hierarchically arranged (primary, secondary, tertiary fibres). No endogenous mineral elements in the skeleton. Choanocyte chambers either diplodal (small, spherical) and eurypylous (large, oval). Mode of reproduction viviparous. Larvae brooded parenchymellae with a posterior ring or cap of long cilia.

**Order Halisarcida Bergquist, 1996**

**Diagnosis **(emended after Bergquist and Cook 2002b). Demospongiae with tubular, branched choanocyte chambers. Larvae brooded parenchymellae (dispherulae) with simple undifferentiated histology, and cilia of uniform length. Absence of fibrous and mineral skeleton. Ectosomal and subectosomal skeleton of highly organised fibrillar collagen.

**Order Verongida Bergquist, 1978**

**Diagnosis **(emended after Bergquist and Cook 2002c). Skeletal network, absent in some genera, without inclusions and with no distinction between primary and secondary fibres. The fibre structure is concentrically laminar surrounding a pith of thin fibrillar material. Taxa lacking skeleton show “peculiar verongid characters” such as the presence of complex brominated tyrosine derivates. Choanocyte chambers either diplodal or eurypylous. Mode of reproduction oviparous, larvae unknown.

### Key to the orders of horny sponges

**Table:** 

1	No spongin fibrous skeleton, no endogenous mineral skeleton; choanocyte chambers tubular, branched	Halisarcida
–	Spongin fibrous skeleton present, no endogenous mineral skeleton	2
2	Mineral exogenous inclusions never present in the skeleton fibres that are concentrically laminar surrounding a pith of thin fibrillar material; elliptic choanocyte chambers in species without skeleton	Verongida
–	Almost constant presence of mineral foreign debris (exogenous inclusions) in the core of some or all skeleton fibres	3
3	Skeleton arranged in a tri-dimensional network of skeleton fibres often cored by exogenous mineral inclusions	Dictyoceratida
–	Skeleton arising from a basal plate; fibres dendritically (tree-shaped) arranged as small adjacent ascending fibres; possible presence of exogenous mineral inclusions	Dendroceratida
N.B. Among Dendroceratida some genera (see key to the genera) show a reticulate fibrous skeleton. To complicate things further, among the Dictyoceratida, the genus *Pleraplysilla* has a dendritic not anastomosing skeleton.		

### Key to families of cave-dwelling horny sponges

**Dendroceratida**

**Table:** 

1	Skeletal fibres dendritically (branched as in a tree) arranged	Darwinellidae
2	Skeletal fibres arrangedin a network	Dictyodendrillidae

**Dictyoceratida**

**Table:** 

1	Thin collagenous filaments with a knob at one tip in addition to the main fibrous skeleton	Irciniidae
–	Lacking filaments	2
2	Homogeneous skeleton fibres, lacking marked laminations	3
–	Primary and secondary fibres with clearly defined laminae	Thorectidae
3	Secondary fibres always lacking inclusions	Spongiidae
–	Primary and secondary fibres packed with by mineral inclusions; spongin frequently scanty, not evident; few species with secondaries partly free of inclusions	Dysideidae

**Halisarcida**

**Table:** 

1	No skeleton	Halisarcidae

**Verongida**

**Table:** 

1	Presence of skeleton	Aplysinidae
2	No skeleton	Ianthellidae

### Key to genera of cave-dwelling horny sponges

**Darwinellidae**

**Table:** 

1	Free, fibrous (horny) spicules (mono- to poly-actines) in the choanosome	*Darwinella*
–	No horny spicules	2
2	Branched, dendritic (not anastomosing) skeleton supporting the erect growth form	3
–	Adjacent fibres dendritically arranged (encrusting growth form)	*Aplysilla*
3Sandy reticulate sponge surface	*Chelonaplysilla*

**Dictyodendrillidae**

**Table:** 

1	Regularly reticulate fibrous skeleton, uncored	*Spongionella*

**Dictyoceratida**

**Dysideidae**

**Table:** 

1	Skeleton of fibres dendritically arranged or free detritus	3
2	Dendritic skeleton (Anastomosed fibres)	4
3	Skeleton of branched (dendritic not anastomosing) tracts of cored spongin	*Pleraplysilla*
4	Primary and secondary fibres cored with mineral detritus	*Dysidea*
–	Primary fibres cored, secondary fibres uncored	*Euryspongia*

**Irciniidae**

**Table:** 

1	Primary fibres often cored with foreign debris	*Ircinia*
–	Primary fibres uncored, or with few inclusions (mainly spicule fragments)	*Sarcotragus*

**Spongiidae**

**Table:** 

1	Surface armoured by foreign debris	*Coscinoderma*
–	Surface unarmoured	2
2	Skeletal network of primary (cored) and secondary (uncored) fibres; large (1-3 cm) lacunae ln the choanosome	*Hippospongia*
–	Skeletal network of primary (cored) and secondary (uncored) fibres; choanosomal lacunae absent	*Spongia*

**Thorectidae**

**Table:** 

1	Laminate skeleton; cored primary and secondary fibres	*Hyrtios*
–	Laminate skeleton; cored primary fibres; secondary fibres free of debris	2
2	Laminate skeleton; primary fibres arranged in single lines	*Cacospongia*
–	Laminate skeleton; fasciculate (grouped) primary fibres	*Fasciospongia*

**Halisarcida**

**Halisarcidae**

**Table:** 

1	No skeleton; smooth, encrusting growth form	*Halisarca*

**Verongida**

**Aplysinidae**

**Table:** 

1	Yellow, massive to digitate growth form; surface reticulate, smooth; skeleton uncored, laminate	*Aplysina*

**Ianthellidae**

**Table:** 

1	Yellow to pink, thin crusts (1-5 mm); surface striate, conulose; skeleton absent	*Hexadella*

### Species descriptions

#### Aplysilla rosea

(Barrois, 1876)

http://species-id.net/wiki/Aplysilla_rosea

Fig. 3

Verongia rosea Barrois, 1876: 57.

##### Description.

Growth form encrusting, thin (3–6 mm), in irregular patches of up to 20 cm in diameter. Surface evidently conulose (1–3 mm) because of the dense dendritic “forest” of “small horny trees” forming the typical skeleton of all *Aplysilla* species. Oscules (1–3 mm) scattered and not evident; inhalant apertures rarely visible *in vivo*. Colour from rose to yellow. Skeleton of large ramified fibres arising from a spongin basal plate strictly adhering to the substratum. Dendritic fibres with maximum size of *ca*. 5 mm in length, *ca*. 300 µm in diameter at the basal portion, and no more than 50 µm in diameter at terminal branches (up to 4–6 sometimes anastomosing). Spongin layered, transparent, pale in colour, not cored with mineral debris.

##### Habitat.

Cave, rocky/detritic/muddy bottom, hyperhaline canal (Manfredonia), artificial reef, coralligenous community, and epibiotic on red coral and on *Pinna nobilis* (L., 1758). Bathymetric range 1–110 m.

##### Mediterranean Caves.

Blava, Calamars, La Catedral, J1 caves (Balearic Sea); Galatea Cave* (Sardinian Sea); Béar, Troc, Endoume, Figuier, Trèmies, Niolon caves (Gulf of Lions); Western-Zoagli Cave (Ligurian Sea); Mago, Gaiola, Secca delle Formiche-Vivara, Mitigliano caves (Central Tyrrhenian Sea); Azzurra Cave (Southern Tyrrhenian Sea); Taccio Vecchio 1 Cave-Lampedusa*, Zembra caves (Sicily Channel); La Regina Cave (Southern Adriatic Sea); Trypia Spilia, Ftelio, Madhes, Andros caves (Aegean Sea) (Vacelet 1959; Sarà 1961a 1964a; Labate 1965; Boury-Esnault 1971; Pouliquen 1972; Pulitzer-Finali and Pronzato 1976, 1980; Pansini et al. 1977; Pulitzer-Finali 1977; Pansini and Pronzato 1982; Balduzzi et al. 1989; Bibiloni et al. 1989; Benedetti-Cecchi et al. 1998; Ben Mustapha et al. 2003; Pronzato and Manconi 2011; Cadeddu 2012; Gerovasileiou and Voultsiadou 2012).

**Figure 3:**
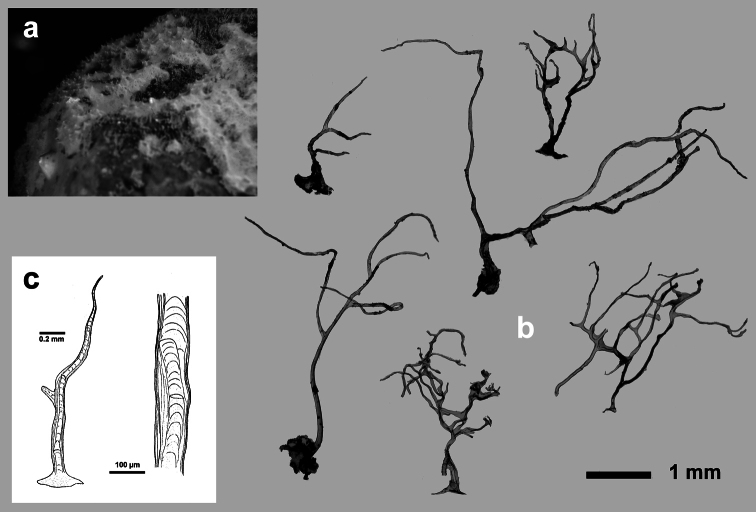
*Aplysilla rosea*. **a** encrusting conulose specimen *ca*. 10 cm in diameter **b** dendritic-arborescent skeleton with ascending spongin fibres of different specimens **c** details of uncored spongin fibres. **c** modified from Vacelet (1959).

#### Chelonaplysilla noevus

(Carter, 1876)

http://species-id.net/wiki/Chelonaplysilla_noevus

Fig. 4

Aplysina noevus Carter, 1876: 229.

##### Description.

Growth form encrusting (less than 2 mm in height). Surface conulose, ornamented by a network of rounded meshes (200–300 µm in diameter) loaded of inclusions; inside the meshes surface is smooth and perforated by small apertures (15–40 µm in diameter). Colour from grey to violet (Vacelet 1959, 1969). Dendritic modules (tree-shaped) of the skeleton with fibres apically branched (80 µm in diameter at their base, 20 µm at the apical branch level).

##### Habitat.

Cave, coralligenous community, rocky bottom. On small pebbles or epibiotic on *Microcosmus vulgaris* Heller, 1877, *Corallium rubrum* (L., 1759) and *Sarcotragus foetidus*. Bathymetric range 1–150 m.

##### Mediterranean caves.

Blava, Calamars, Misidacis caves (Balearic Sea); Endoume, Figuier, Trèmies caves (Gulf of Lions) (Pouliquen 1972; Uriz et al. 1992; Martì et al. 2004; Pronzato and Manconi 2011).

**Figure 4:**
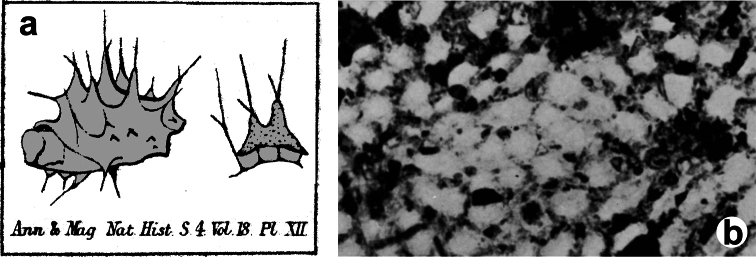
*Chelonaplysilla noevus*. **a** original illustration of the type specimen encrusting with conulose surface **b** close-up of the sponge surface with mineral debris and smooth rounded inhalant areas (lighter in the scheme) bearing small ostia; **a** modified from Carter (1876) **b** modified from Topsent (1925).

#### Darwinella simplex

Topsent, 1892

http://species-id.net/wiki/Darwinella_simplex

Fig. 5

Darwinella simplex Topsent, 1892: 27.

##### Description.

Growth form encrusting. Surface conulose bearing a reticulate dermal membrane with fibre tips supporting conules. Colour *in vivo* “rouge carmin” as reported by the author, bright red. Dendritic skeleton arising from a basal spongin plate with the main fibres (up to 4 mm in height, 60–160 µm in diameter) evidently laminated and free of foreign material, with variably dense granular axial pith. Fibres. Horny spicules triactines free or connected to the main skeleton (rarely each to one another), with actins *ca*. 1.1–1.25 mm in length and 45–50 µm in diameter, gradually tapering towards the sharp tips. Rays linear, usually 3, rarely 2 or 4. Spicules sometimes with pith.

##### Habitat.

Cave, rocky bottom, coralligenous community. Bathymetric range 3–100 m.

##### Mediterranean caves.

Lerici Cave (Ligurian Sea); Secca delle Formiche-Vivara Cave (Central Tyrrhenian Sea); Taccio Vecchio 1 Cave-Lampedusa* (Sicily Channel) (Pulitzer-Finali and Pronzato 1976, 1980; Pronzato and Manconi 2011). Recorded as *Darwinella australiensis*.

##### Remarks.

Pronzato (1975) considered the Mediterranean species *Darwinella simplex* Topsent, 1892 as junior synonym of the Pacific species *Darwinella australiensis* Carter, 1885 (senior synonym) sharing diagnostic morphological traits as also focused by Topsent (1892). A re-evaluation of original descriptions vs. old and new materials allow us to consider *Darwinella simplex* Topsent, 1892 a valid species. The validity of *Darwinella simplex* solves the extremely disjunct Australian-Mediterranean geographic pattern and matches the hypothesis of a species complex.

**Figure 5:**
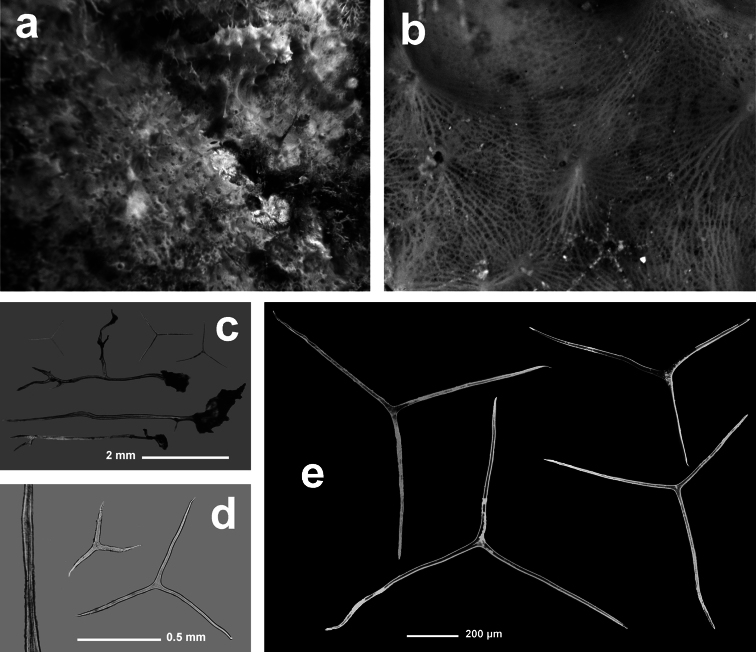
*Darwinella simplex*. **a** encrusting specimen *in vivo* (*ca*. 10 cm in diameter) **b** close up of the sponge surface bearing a reticulate dermal membrane with primary fibre tips supporting conules **c, d** laminate spongin fibre (free of foreign material) and free horny spicules (LM) **e** free horny spicules (SEM).

#### Spongionella gracilis

(Vosmaer, 1883)

http://species-id.net/wiki/Spongionella_gracilis

Fig. 6

Velinea gracilis Vosmaer, 1883: 439.

##### Description.

Tubular habit with ten to fifteen slightly clavate hollow cylinders (up to 2 cm high, with a diameter of 5–8 mm) partly coalescing and arising from a common basal spongin plate (*ca*. 4.5 × 3 cm in diameter). Consistency soft and elastic, as the rule in all *Spongionella* species. Oscules apical (2–3 mm in diameter). Surface finely conulose with conules supported by tips of ascending fibres (conules *ca*. 100 µm high, 300 µm apart). Skeleton reticulate with a more or less regular network of generally quadrangular meshes (100–300 µm in diameter). Primary fibres (25–30 µm in diameter) connected by rare and irregular tracts (5–10 µm in diameter). Fibres laminated, clear, and uncored, with a transparent axis.

##### Habitat.

Cave, rocky bottom, epibiotic on *Corallium rubrum*. Bathymetric range 9–45 m.

##### Mediterranean Caves.

Secca delle Formiche–Vivara Cave (Central Tyrrhenian Sea) (Pulitzer-Finali and Pronzato 1976, 1980; Pulitzer-Finali 1977; Pronzato and Manconi 2011).

##### Remarks.

The reticulate fibrous skeleton is atypical for Dendroceratida.

**Figure 6:**
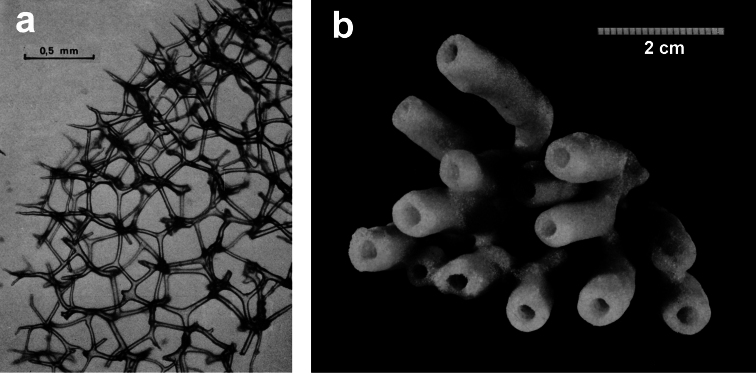
*Spongionella gracilis*. **a** typical regular arrangement of the very clear uncored fibres in the skeletal network **b** a preserved digitate specimen. Modified from Pulitzer-Finali and Pronzato (1980).

#### Spongionella pulchella

(Sowerby, 1804)

http://species-id.net/wiki/Spongionella_pulchella

Fig. 7

Spongia pulchella Sowerby, 1806: 87.

##### Description.

Growth form of Mediterranean specimens cushion-like, small (2 cm in diameter, 5–10 mm in thickness). Colour grey-greenish-brown. Consistency soft and elastic. Surface finely conulose with conules supported by tips of ascending fibres. Inhalant apertures not visible, oscules small (0.5–1 mm) and rare. Flagellate chambers large (70–80 µm) with small choanocytes. Skeleton network typical of the genus, extremely regular and practically indistinguishable from that of *Spongionella gracilis*. Fibres laminate, light and transparent, with axial pith lacking of inclusions that, when evident, shows a typical aplysillid structure. After Topsent (1929): primary fibres of a single dimensional class (25–35 µm); rare and irregular secondary connecting tracts (7–25 µm); meshes generally quadrangular 120–300 µm in diameter.

##### Habitat.

Cave, coralligenous community, *Posidonia oceanica* meadow, artificial reef, detritic bottom. Bathymetric range 4–380 m.

##### Mediterranean Caves.

Meda Petita, Petita de la Vaca caves (Balearic Sea); Endoume, Figuier, Trèmies caves (Gulf of Lions); Farà Cave (Aegean Sea) (Pouliquen 1972; Bibiloni et al. 1984a; Pronzato and Manconi 2011; Gerovasileiou and Voultsiadou 2012).

##### Remarks.

The Mediterranean specimens ascribed to this species, are very different from the Atlantic ones.

**Figure 7:**
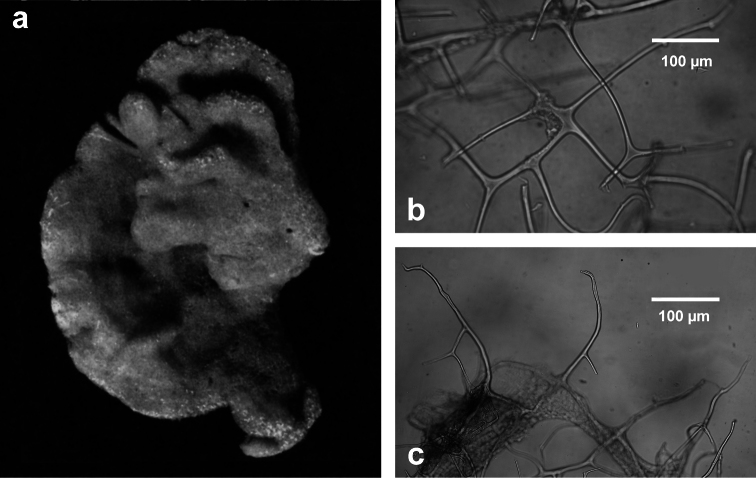
*Spongionella pulchella*. **a** the specimen described by Topsent **b** choanosomal skeleton (LM) **c** tips of fibres at the sponge surface (LM). a) modified from Topsent (1901).

#### Dysidea avara

(Schmidt, 1862)

http://species-id.net/wiki/Dysidea_avara

Fig. 8

Spongelia avara Schmidt, 1862: 29.

##### Description.

Growth form usually irregularly massive (2–4 cm large, 1–2 cm thick) and commonly lobate. Specimens with large size (15–20 cm in diameter) and long digitations (5 cm) not infrequent. Colour constantly light rose-violet. Surface free of foreign debris, conulose with a regular fibrous network interconnecting apices of conules; conules large (3–6 mm high, 2–6 mm apart, sometimes clubbed). Oscules (4–10 mm in diameter) apical on digitations with a very delicate transluscent collar (2–4 mm) sometimes evident in living specimens; inhalant apertures (30–50 µm in diameter) scattered. Choanosome lax with ovoid choanocyte chambers (70 µm in diameter). Skeleton as a three-dimensional network of irregular polygonal meshes (100–800 µm) with primary fibres extremely variable in size (60–300 µm) constantly and heavily filled by foreign material; secondary ones (20–40 µm) with light and laminated spongin almost regularly free of debris or with scattered grains. Reproduction reported in June.

##### Habitat.

Cave, coralligenous community, artificial reefs, rocky/muddy/detritic bottom, lagoon, *Posidonia oceanica* meadow. Bathymetric range 1–100 m.

##### Mediterranean caves.

Blava, Meda Petita, Petita de la Vaca, Blue, Misidacis caves (Balearic Sea); Galatea*, Falco*, Bisbe* caves (Sardinian Sea); Béar, Troc, Endoume caves (Gulf of Lions), Bergeggi Cave (Ligurian Sea); Taccio Vecchio 1 Cave-Lampedusa* (Sicily Channel); Sifone Cave (Ionian Sea); Croatian, Columbera, Stražica caves (Northern Adriatic Sea); Sorrentino, Spido, Bue Marino caves (Southern Adriatic Sea); Farà Cave (Aegean Sea) (Boury-Esnault 1971; Pouliquen 1972; Pulitzer-Finali and Pronzato 1980; Bibiloni et al. 1984a, b; Bianchi and Morri 1994; Corriero et al. 2000; Novosel et al. 2002; Martì et al. 2004; Faresi et al. 2006; Turon et al. 2009; Denitto et al. 2010; Pronzato and Manconi 2011; Bakran-Petricioli et al. 2012; Cadeddu 2012; Gerovasileiou and Voultsiadou 2012).

**Figure 8:**
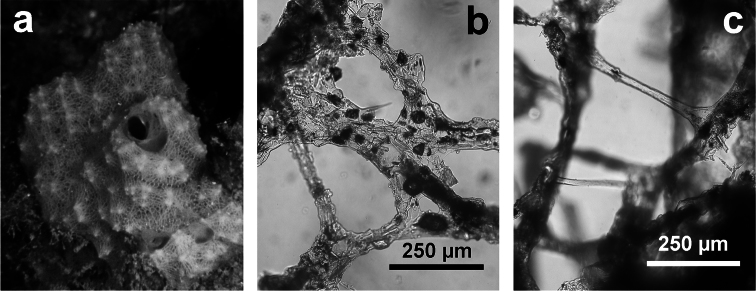
*Dysidea avara*. **a** massive specimen (*ca*. 5 cm in diameter) showing a large osculum **b, c** the skeletal network with primary (cored) and secondary (almost uncored) fibres.

#### Dysidea fragilis

(Montagu, 1818)

http://species-id.net/wiki/Dysidea_fragilis

Fig. 9

Spongia fragilis Montagu, 1818: 114.

##### Description.

Growth form irregular, massive; usually less than 10 cm in diameter, sometimes up to 15–20 cm in diameter and 2–3 cm in height. Colour *in vivo* (generally also preserved specimens) light grey to white; several, slightly perceptible, tone dominances are possible (light green to light brown). Consistency soft and fragile. Surface, shared by all species of the genus, as an irregular network of dense collagen fibres, sometimes with mineral debris. Inhalant apertures 80–120 µm in diameter. Oscules scattered (2–4 mm in diameter). Light collagen amount (fibrous reticulate) in the mesohyl. Flagellate chambers large. Skeleton reticulate, with irregular meshes (300–600 µm), and extremely fragile because of scanty spongin and extreme abundance of mineral granulation. Primary and secondary fibres (40–200 µm) not distinguishable or hierarchically organized.

##### Habitat.

Cave, rocky/detritic/muddy/sandy bottom, coralligenous community, *Posidonia oceanica* meadow, lagoon, artificial reefs, epibiotic on *Pinna nobilis*. Bathymetric range 1–200 m.

##### Mediterranean caves.

La Catedral, Tunel LLarg, Petita de la Vaca caves (Balearic Sea); Galatea*, Falco*, Bisbe* caves (Sardinian Sea); Béar, Niolon caves (Gulf of Lions); western-Zoagli, Piccola Zoagli-Chiavari, Tunnel Zoagli-Chiavari, Eastern Bonassola caves (Ligurian Sea); Mago, Gaiola, Misteri, Tuffo Tuffo, Mitigliano caves (Central Tyrrhenian Sea); Infreschi Cave (Southern Tyrrhenian Sea); Taccio Vecchio 1 Cave-Lampedusa*, Tunnel of Cani Islands (Sicily Channel); Gamberi* Cave (Ionian Sea); Croatian caves (Northern Adriatic Sea); La Regina Cave (Southern Adriatic Sea); Farà Cave (Aegean Sea) (Vacelet 1959; Sarà 1961a, 1962, 1964a; Labate 1964, 1965; Rützler 1966; Boury-Esnault 1971; Pulitzer-Finali and Pronzato 1976; Pansini et al. 1977; Pulitzer-Finali 1977; Bibiloni et al. 1984b, 1989; Ben Mustapha et al. 2002; Pronzato and Manconi 2011; Bakran-Petricioli et al. 2012; Cadeddu 2012; Gerovasileiou and Voultsiadou 2012).

**Figure 9:**
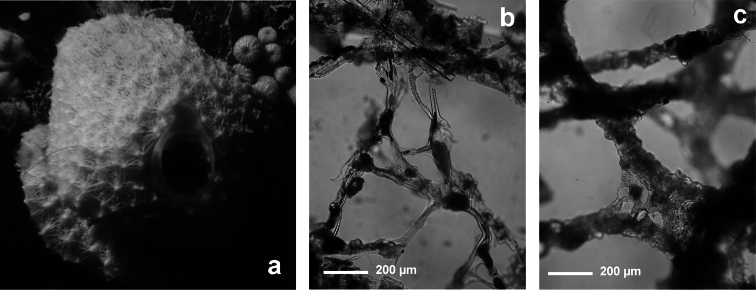
*Dysidea fragilis*. **a** massive specimen (*ca*. 3 cm in diameter) with an apical osculum; **b, c** reticulate skeletal network and irregular meshes of primary and secondary fibres with scanty spongin (LM).

#### Dysidea incrustans

(Schmidt, 1862)

http://species-id.net/wiki/Dysidea_incrustans

Fig. 10

Spongelia incrustans Schmidt, 1862: 29.

##### Description.

Growth form encrusting (3–8 mm thick). Consistency fragile. Colour light grey to pale violet. Surface reticulate, conulose showing the internal aquiferous system in transparency. Conules 1–3 mm high, 3–5 mm apart. Oscules (5–7 mm) scattered, with a transparent collar. Skeletal network irregular with meshes (200–600 µm in diameter) formed by ascending primary fibres (70–90 µm in diameter) cored of foreign material, and secondary fibres (5–30 µm in diameter) generally lacking inclusions.

##### Habitat.

Cave, rocky bottom, artificial reefs, *Posidonia oceanica* meadow, lagoon, also. Frequently as encrusting patches also on other sponges or epibiotic on *Pinna nobilis*. Bathymetric range 1–100 m.

##### Mediterranean Caves.

Galatea* Cave (Sardinian Sea); Lerici Cave (Ligurian Sea); Mago, Mitigliano caves (Central Tyrrhenian Sea); Taccio Vecchio 1 Cave-Lampedusa* (Sicily Channel); Gamberi*, Gymnasium* caves (Ionian Sea) (Pulitzer-Finali and Pronzato 1976, 1980; Pansini et al. 1977; Pulitzer-Finali 1977; Pansini and Pronzato 1982; Pronzato and Manconi 2011; Cadeddu 2012).

**Figure 10:**
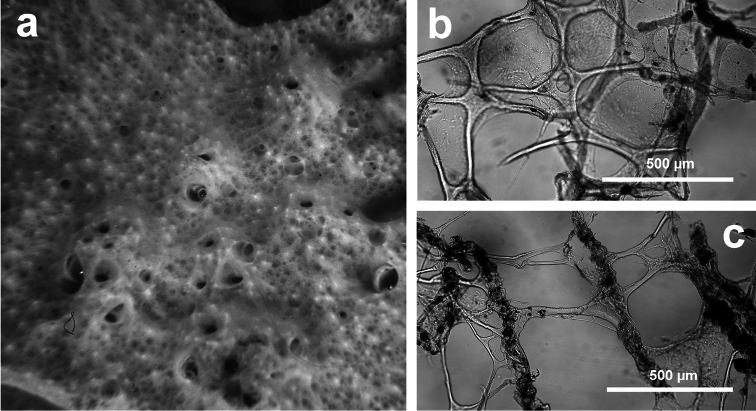
*Dysidea incrustans*. **a** close up of a large (*ca*. 20 cm) encrusting specimen showing scattered small oscula and visible inhalant pores **b** reticulate skeleton with a secondary network of slimmer fibres almost free of inclusions **c** main fibres cored of foreign material supporting the conules at the sponge surface.

#### Dysidea tupha

(Martens, 1824)

http://species-id.net/wiki/Dysidea_tupha

Fig. 11

Spongia tupha Martens, 1824: 534.

##### Description.

Growth form as a meshed irregular network of cylindrical processes (8–10 cm in length, 05–1 cm in diameter) lying on the substratum, rarely erected in some parts. Colour whitish to pale-light brown. Surface finely and irregularly conulose (0.3–1 mm high and apart). Oscules small (1 mm) and irregularly scattered. Skeleton network with irregular or quadrangular meshes (*ca*. 0.5 mm) with ascending primary fibres (80–120 µm) supporting conules. Primaries moderately charged of mineral materials; secondary fibres slim (15–40 µm) and almost free of sand grains.

##### Habitat.

Cave, rocky/detritic/muddy bottom, coralligenous community, lagoon. Bathymetric range 1–450 m.

##### Mediterranean caves.

Mitigliano Cave (Central Tyrrhenian Sea); Tunnel of Cani Islands, Tunnel of Tabarka (Sicily Channel) (Balduzzi et al. 1989; Ben Mustapha et al. 2002, 2003; Pronzato and Manconi 2011).

**Figure 11:**
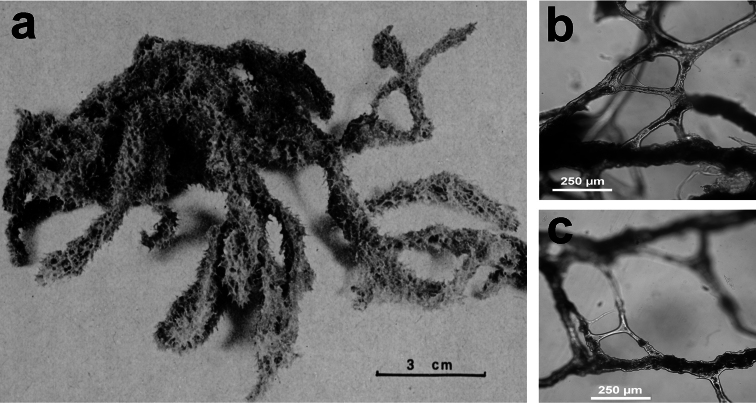
*Dysidea tupha*. **a** specimen with typical cylindrical processes and finely, irregularly conulose surface; **b, c** views of the skeleton with fibres variably charged of mineral detritus (LM).

#### Euryspongia raouchensis

Vacelet, Bitar, Carteron, Zibrowius and Perez, 2007

http://species-id.net/wiki/Euryspongia_raouchensis

Fig. 12

Euryspongia raouchensis Vacelet, Bitar, Carteron, Zibrowius & Perez, 2007: 1548

##### Description.

Growth form encrusting (6 × 4 cm, *ca*. 3–5 mm thick). Surface covered of small conules (0.8–1.2 mm apart) each with a slightly protruding fibre. Ectosome unarmoured. Oscules (0.8–1 mm in diameter) numerous, circular and irregularly scattered. Colour cream *in vivo* with the tips of conules whitish, clear brown in alcohol. Consistency fleshy, easily torn. Choanocyte chambers of the dysideid type, numerous, large (75–90 µm in diameter). Skeleton primary fibres heavily cored (125–150 µm in diameter), ascending singly from substratum to surface, rather regularly spaced, ending as conules. Secondary fibres (40–70 µm in diameter) generally clear of inclusions can have a poorly developed central core of foreign material.

##### Habitat.

Cave. Exclusively known from Raouché cave, along the Lebanese coast (Eastern Mediterranean Sea). Bathymetric distribution 2–5 m.

##### Mediterranean caves.

Raouché Cave (Levantine Basin) (Vacelet et al. 2007; Pronzato and Manconi 2011).

**Figure 12:**
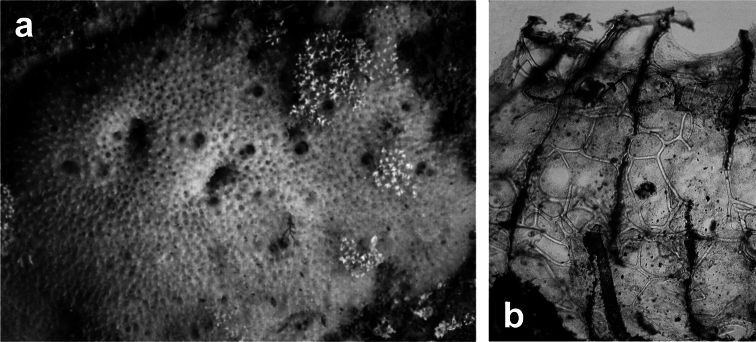
*Euryspongia raouchensis*. **a** underwater image of a living specimen **b** small conules (thin section by LM) with slightly protruding fibres at the sponge surface and skeletal network with cored ascending primaries and uncored secondaries. **a, b** modified from Vacelet et al. (2007).

#### Pleraplysilla minchini

Topsent, 1905

http://species-id.net/wiki/Pleraplysilla_minchini

Fig. 13

Pleraplysilla minchini Topsent, 1905: 184.

##### Description.

Growth form encrusting (1–5 mm in thickness). Consistency soft. Colour light brown to light grey. Surface finely conulose. Exhalant canals evident on the sponge surface, converging in scattered oscules 1–2 mm in diameter. Flagellate chambers from oval to rounded (50–90 µm in diameter). Skeleton typically dendritic with fibres (1–3 mm in height *ca*. 160 µm in diameter at their base) rising from a basal plate. Fibres laminated, normally with a single apex supporting a conule but, in some cases, arborescent with 2–3 branches. Fibres evidently cored with irregularly dense foreign debris, mainly spicule fragments.

##### Habitat.

Cave, rocky bottom, artificial reefs. Bathymetric range 1–30 m.

##### Mediterranean caves.

Niolon Cave (Gulf of Lions); Monte Vico, Secca delle Formiche-Vivara, Mago caves (Central Tyrrhenian Sea) (Laborel and Vacelet 1958; Pulitzer-Finali and Pronzato 1976; Pansini et al. 1977; Pulitzer-Finali 1977; Pronzato and Manconi 2011).

##### Remarks.

As for diagnostic traits the genus *Pleraplysilla* is anomalous among the Dictyoceratida, for the trait ‘dendritic not anastomosing skeleton’. As for the taxonomic status *Pleraplysilla minchini* is regarded by Vacelet (1959) as a synonym of *Pleraplysilla spinifera*. Later authors, as Cabioch (1968) and Borojevic et al. (1968), considered both species as valid. The material available for our study seems to confirm a specific divergence between the two. *Pleraplysilla spinifera* is generally recognizable at sight by the very pronounced, spaced conules. Its fibres reach a length of 12 mm, with a thickness of 450 µm near the base; they are generally branched; sometimes more than one fibre starts from a common basal plate; the inclusions are mostly closely-packed sand grains. In *Pleraplysilla minchini* the fibres are less widely spaced, they reach not more than 3 mm in length and a diameter of 160 µm near the base; they are generally not branched and there is a prevalence of sponge spicules in their inclusions.

**Figure 13:**
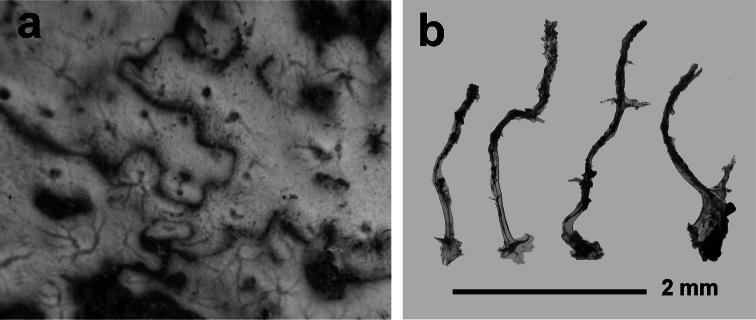
*Pleraplysilla minchini*. **a** encrusting specimens in a small facies (Mitigliano Cave) **b** detail of dendritic skeleton fibres with debris filling the axial core.

#### Pleraplysilla spinifera

(Schulze, 1878)

http://species-id.net/wiki/Pleraplysilla_spinifera

Fig. 14

Spongelia spinifera Schulze, 1878b: 152.

##### Description.

Growth form encrusting, up to 2 cm thick, as irregular patches (several cm in diameter) characterized by a smooth and conulose mucous surface. Conules very evident, up to 8–10 mm in height. Colour from whitish to very light brown. Consistency very soft. Exhalant and inhalant apertures (up to 1 mm in diameter) irregularly scattered on the surface. Skeleton of dendritic fibres generally arborescent with 2–5 branches. Each fibre with a basal plate strictly adhering to the substrate. Spongin laminated and cored by sand grains and spicule fragments. These stout fibres (1.5–2.0 mm in height) can reach 400 µm in diameter at their base, with a sandy core of 80 µm. Fibres usually light yellow and transparent show, in many cases, a red-brown colour due to microscopic algae.

##### Habitat.

Cave, rocky/detritic/muddy bottom, red coral bank, coralligenous community, artificial barriers, boulders, *Posidonia oceanica* meadow. In many cases massive specimens, not over 5 cm in diameter, of this species are epibiotic on gorgonians and *Pinna nobilis*. Bathymetric range 1–500 m.

##### Mediterranean caves.

Blava, La Catedral, Blu, Misidacis, Meda Petita, Petita de la Vaca caves (Balearic Sea); Galatea*, Falco*, Bisbe* caves (Sardinian Sea); Béar, Endoume, Figuier, Tremier, Niolon, Bagaud caves (Gulf of Lions); Secca delle Formiche –Vivara Cave (Central Tyrrhenian Sea); Gamberi* Cave (Ionian Sea); Croatian caves (Northern Adriatic Sea); Piccolo Ciolo, Marinella, Principessa caves (Southern Adriatic Sea); Farà, Agios Vasilios, Vouliagnemi caves (Aegean Sea) (Vacelet 1959; Boury-Esnault 1971; Pouliquen 1972; Pulitzer-Finali and Pronzato 1976; Pulitzer-Finali 1977; Bibiloni et al. 1984a, 1989; Harmelin et al. 2003; Martì et al. 2004; Bussotti et al. 2006; Turon et al. 2009; Pronzato and Manconi 2011; Bakran-Petricioli et al. 2012; Cadeddu 2012; Gerovasileiou and Voultsiadou 2012).

##### Remarks.

Among the Dictyoceratida, the genus *Pleraplysilla* has a dendritic not anastomosing skeleton.

**Figure 14:**
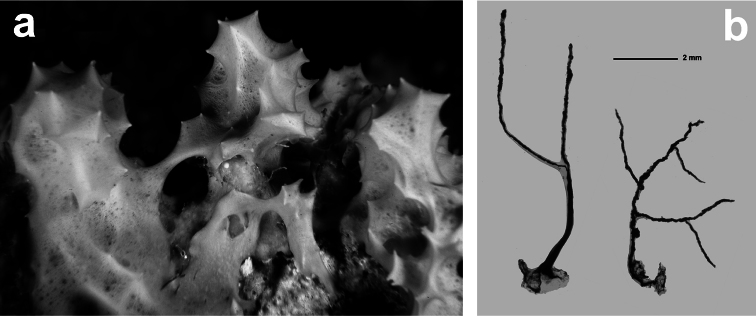
*Pleraplysilla spinifera*. **a** large specimen (*ca*. 5 cm) **b** ramified, cored dendritic fibres (LM).

#### Ircinia dendroides

(Schmidt, 1862)

http://species-id.net/wiki/Ircinia_dendroides

Fig. 15

Hircinia dendroides Schmidt, 1862: 32, 1868.

##### Description.

Growth form partially erect (*ca*. 5–10 cm in diameter) with quite cylindrical ramifications (0.8–1.5 cm in thickness) anastomosing in a lax irregular network growing flat on the substrate with few short uprising processes. Colour light to dark grey. Consistency finely sandy. Inhalant and exhalant apertures not evident. Skeleton network irregularly reticulate with large meshes (100–500 µm in diameter) of primary (120–200 µm) and secondary (30–90 µm) fibres. Primaries with a dark pith rich of foreign inclusions; secondaries laminated and converging in several cribrose plates. Spongin filaments abundant (3.5–5.0 µm thick), with a terminal knob (8–10 µm).

##### Habitat.

Cave, detritic and rocky bottom, coralligenous community. Bathymetric range 1–110 m.

##### Mediterranean caves.

Blava, Calamars, La Catedral, Meda Petita, Petita de la Vaca, Blue, Misidacis caves (Balearic Sea); Bagaud Cave (Gulf of Lions); Azzurra, Mago, Misteri caves (Central Tyrrhenian Sea); Taccio Vecchio 1 Cave-Lampedusa* (Sicily Channel); Castro Marina, Mazzere*, Gamberi*, Gymnasium* Caves (Ionian Sea); Croatian, Stražica caves (Northern Adriatic Sea); Viole, Spido caves (Southern Adriatic Sea); Agios Nicolaos Cave (Aegean Sea) (Pansini et al. 1977; Pulitzer-Finali and Pronzato 1980; Bibiloni et al. 1984a, b, 1989; Uriz et al. 1992; Novosel et al. 2002; Harmelin et al. 2003; Pronzato and Manconi 2011; Bakran-Petricioli et al. 2012).

**Figure 15:**
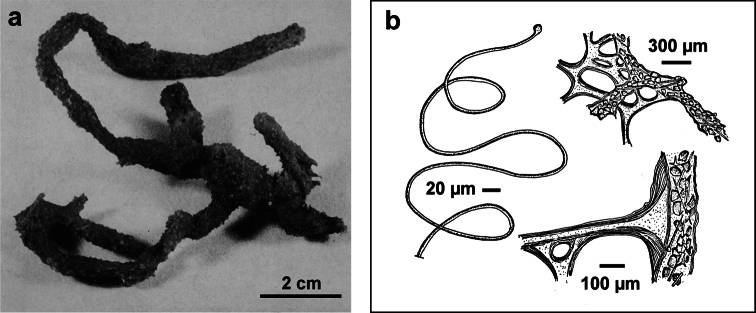
*Ircinia dendroides*. **a** specimen with typical cylindrical ramifications **b** details of the skeletal network with cored primary fibres, uncored secondaries forming large cribrose plates, and filaments with the typical apical knob. **a** modified from Pulitzer-Finali and Pronzato (1980) **b** modified from Rubió-Lois et al. (1981).

#### Ircinia oros

(Schmidt, 1864)

http://species-id.net/wiki/Ircinia_oros

Fig. 16

Hircinia oros Schmidt, 1864: 29.

##### Description.

Growth form massive, lobate, with large size (20-30 cm in diameter and 10–15 in height). Each lobe usually bears a large oscule (30–60 mm in diameter), sometimes at the end of a short funnel (1 cm high). Colour medium to dark grey in vivo. Surface covered by a slim layer of very fine and regular mineral sediment engulfed in a slender regular network showing a lighter colour. Conules (1–2 mm in height) regularly distributed, 24 mm apart. Choanosomal skeleton rust coloured and rich in fibres and filaments. Skeleton network of cored primary fibres (200–250 µm in diameter) and free (or almost free) secondary fibres (100–200 µm). Filaments (9–13 µm) with an oval knob (15–22 µm).

##### Habitat.

Cave, detritic and rocky bottom, coralligenous community. Specimens of this species are frequently covered by large specimens of *Haliclona (Reniera) cratera* (Schmidt 1862). Bathymetric range 1–150 m.

##### Mediterranean caves.

Blava, La Catedral, J1, Blue, Misidacis caves (Balearic Sea); Galatea*, Falco*, Bisbe* caves (Sardinian Sea); Endoume, Figuiers caves (Gulf of Lions); Western-Zoagli Cave (Ligurian Sea); Lacco Ameno, Tuffo Tuffo caves (Central Tyrrhenian Sea); Monastir, Salakta caves (Sicily Channel); Mazzere* Cave (Ionian Sea); Croatian caves (Northern Adriatic Sea); Trypia Spilia, Ftelio, Farà, Madhes, Alikes caves (Aegean Sea) (Sarà 1960a, 1964a; Rützler 1966; Pouliquen 1972; Bibiloni et al. 1989; Ben Mustapha et al. 2003; Martì et al. 2004; Turon et al. 2009; Pronzato and Manconi 2011; Bakran-Petricioli et al. 2012; Cadeddu 2012; Gerovasileiou and Voultsiadou 2012).

**Figure 16:**
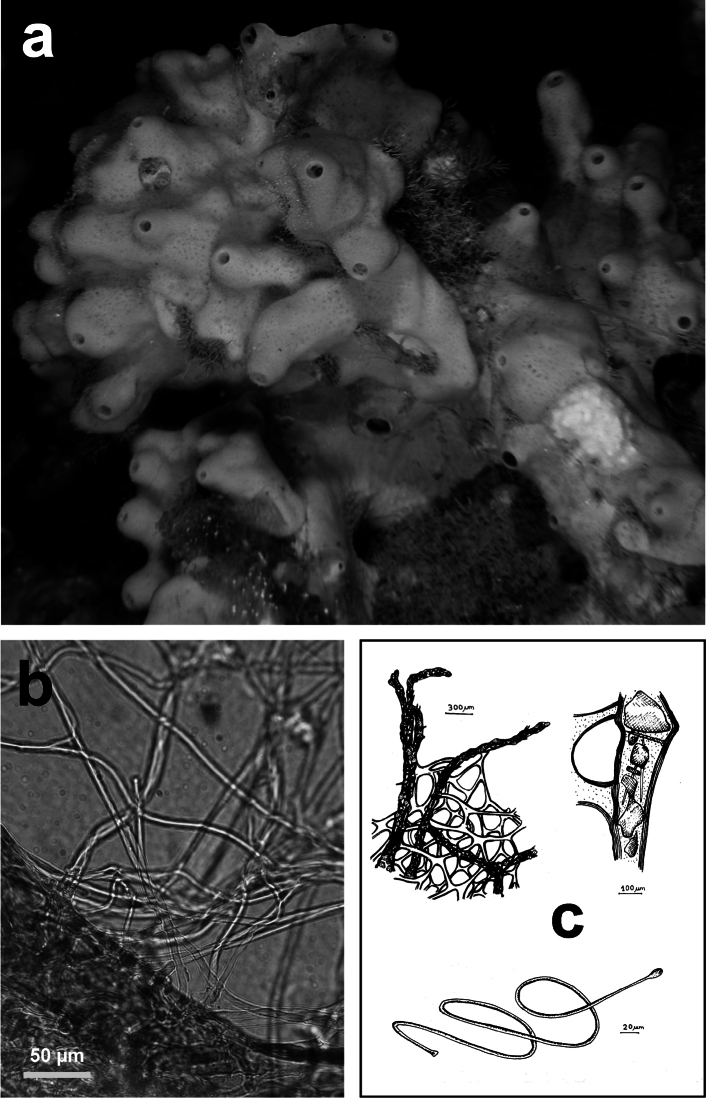
*Ircinia oros*. **a** specimen with an epibiotic haliclonid (lightest area) **b** magnifications (LM) of typical irciniid skeletal filaments **c** schematic drawings of cored primary fibres, uncored secondary network and a filament with the terminal knob. **c** modified from Rubió-Lois et al. (1981).

#### Ircinia paucifilamentosa

Vacelet, 1961

http://species-id.net/wiki/Ircinia_paucifilamentosa

Fig. 17

Ircinia paucifilamentosa Vacelet, 1961a: 354.

##### Description.

This specie was described on behalf of two fragments of “an irregular massive specimen with osculiferous lobes”. Conules few, irregularly high and scattered. Colour reported as “light” in alcohol. Consistency lax, similar to *Cacospongia* species. Dermal membrane reinforced by rare sand grains, easy to remove. Skeleton network of primary fibres cored and anastomosed with secondaries free of foreign materials (dimensions not reported in the original description). Filaments very rare (9–13 µm in diameter) with an irregular globular termination (25–45 µm in diameter). Flagellate chambers 25–35 µm in diameter.

##### Habitat.

Cave. Bathymetric range 1–3 m.

##### Mediterranean caves.

Only known from a few caves in the Aegean Sea at Kastelorizo (type locality), Trypia, Farà and Agios Vasilios caves (Vacelet 1961a; Voultsiadou-Koukoura and Koukouras 1993; Pronzato and Manconi 2011; Gerovasileiou and Voultsiadou 2012).

**Figure 17:**
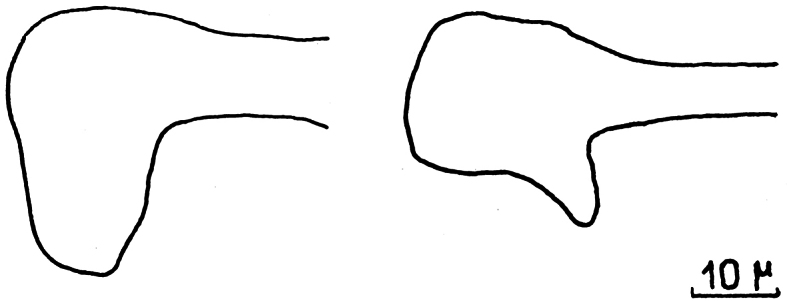
*Ircinia paucifilamentosa*. Peculiar shape of the terminal knobs of filaments in the only available illustration for this species. Modified from Vacelet (1961).

#### Ircinia retidermata

Pulitzer-Finali and Pronzato, 1980

http://species-id.net/wiki/Ircinia_retidermata

Fig. 18

Ircinia retidermata Pulitzer-Finali and Pronzato, 1980: 150.

##### Description.

Growth form massive, rounded, *ca*. 10 × 5 × 5 cm. Consistency firm and elastic. Colour in the preserved state is from beige to mid brown; living specimens appear a little bit darker. Surface conulose with blunt conules (*ca*. 1–2 mm high, 1–3 mm apart) connected with each other by a raised, honeycombed reticulation with meshes (*ca*. 80 µm in diameter) quite conspicuous at bare eye, made of fine particles of sand and a concentration of filaments. Oscules (2–5 mm in diameter) scattered, with elevated margins. Skeleton reticulate with meshes 200 to 600 µm in diameter. Main fibres (50–80 µm in thickness) not fasciculate, moderately cored by foreign matter (sand and spicule fragments). Secondary fibres (20–80 µm thick) irregularly trellis-like, free of inclusions. Filaments *ca*. 5 µm thick.

##### Habitat.

Cave, muddy and rocky bottom. Here we report a new record from a submerged cave in the NW-Sardinian karst. Bathymetric range shallow water up to 80 m.

##### Mediterranean caves.

Falco* Cave (Sardinian Sea) (Cadeddu 2012).

**Figure 18:**
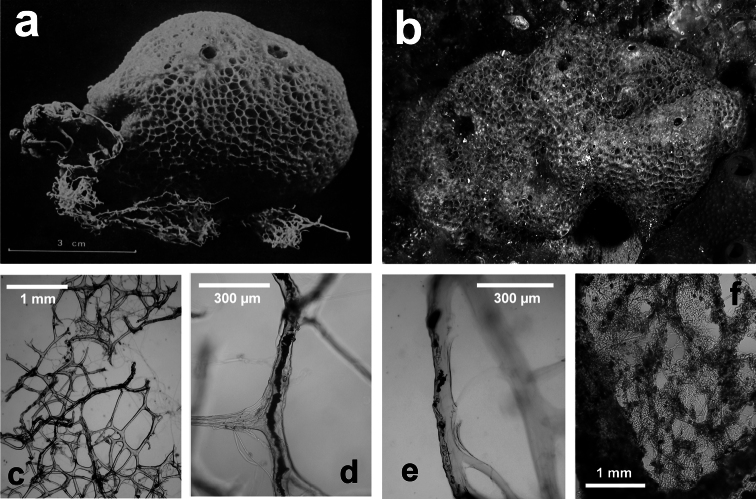
*Ircinia retidermata*. **a** habitus of the type specimen **b** an underwater image of a living specimen **c, d, e** different magnifications (LM) of the skeletal network showing cored primary fibres, uncored secondaries, and the typical irciniid filaments **f** sponge surface finely granulate by mineral debris embedded in a very close fibrillar network. **a** modified from Pulitzer-Finali and Pronzato (1980).

#### Ircinia variabilis

(Schmidt, 1862)

http://species-id.net/wiki/Ircinia_variabilis

Fig. 19

Hircinia variabilis Schmidt, 1862: 34.

##### Description.

Growth form massive up to 20–25 cm in height and diameter. Colour also notably variable: from light or dark grey, to light or dark brown and light or dark violet. Consistency elastic and strong. Dimension and density of conules variable, not representing a valid diagnostic character. Oscules arranged in disorder. Skeleton network of primary (150–250 µm) fibres cored by opaque foreign materials supporting conules at their apices; secondary fibres mostly free of inclusions, and highly variable in diameter (10–200 µm).

##### Habitat.

Cave, coralligenous community, detritic and rocky bottom, *Posidonia oceanica* meadow, lagoon, epibiotic on *Pinna nobilis*. Bathymetric range 0–450 m.

##### Mediterranean caves.

Blava, Blue, Meda Petita, Petita de la Vaca caves (Balearic Sea); Galatea*, Falco*, Bisbe* caves (Sardinian Sea); Niolon Cave (Gulf of Lions); Punta Manara, Western-Bonassola caves (Ligurian Sea); Azzurra, Isolotto, Giannutri, Ponza, Monte Vico, Mago, Secca delle Formiche-Vivara, Misteri, Scraio-Vico Equense, Mitigliano caves (Central Tyrrhenian Sea); Maratea, Azzurra, Leone caves (Southern Tyrrhenian Sea); Taccio Vecchio 1 Cave-Lampedusa* (Sicily Channel); Castro Marina, Porto Cesareo, Mazzere*, Gymnasium* caves (Ionian Sea); Croatian, Vrbnik-Krk, Columbera caves (Northern Adriatic Sea); Pagliai, Viole, Bue Marino, Regina, Torre Incine, Piccolo Ciolo, Marinella, Principessa caves (Southern Adriatic Sea); Gournia Cave (Crete, Aegean Sea) (Vacelet 1959; Sarà 1962, 1964a; Labate 1965; Pulitzer-Finali and Pronzato 1976, 1980; Pansini et al. 1977; Pulitzer-Finali 1977; Pansini and Pronzato 1982; Bibiloni et al. 1984a, b; Balduzzi et al. 1989; Corriero et al. 2000, 2004; Arko-Pjevac et al. 2001; Martì et al. 2004; Bussotti et al. 2006; Faresi et al. 2006; Turon et al. 2009; Pronzato and Manconi 2011; Bakran-Petricioli et al. 2012; Cadeddu 2012).

**Figure 19:**
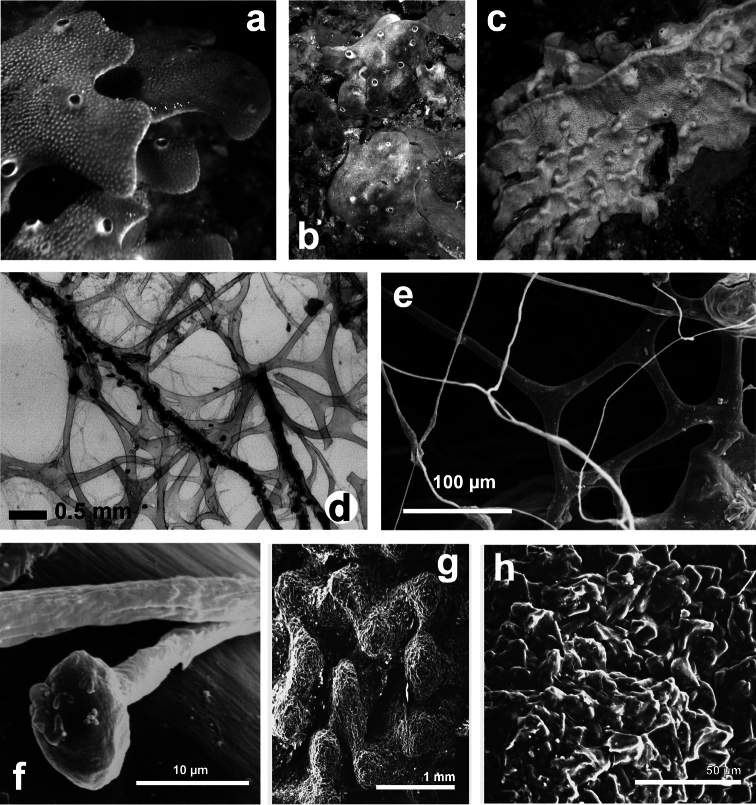
*Ircinia variabilis*. **a–c** wide array of growth forms in different specimen **d** skeletal spongin network of primary and secondary fibres, and filaments (LM) **e** skeletal spongin network of primary and secondary fibres, and filaments (SEM) **f** magnification of a filament at the terminal knob; **g, h** regularly and finely sandy sponge surface. d) modified from Pronzato et al. (2004).

#### Sarcotragus fasciculatus

(Schmidt, 1862) comb. n.

http://species-id.net/wiki/Sarcotragus_fasciculatus

Fig. 20

Hircinia fasciculata Schmidt, 1862: 34

##### Description.

Growth form massive, irregular (up to 12 × 15 cm in diameter). Surface regularly conulose (1 mm in height, 1–2 mm apart). Skeleton network light brown, fragile, reticulate with more or less square meshes from the sponge base to the surface. Almost parallel ascending primary fibres (200–300 µm in diameter) free from foreign inclusions, with apices supporting conules. Each primary fibre as a bundle of some (2–5) uncored secondary fibres (50–100 µm in diameter) joined by conspicuous spongin tracts and cribrose plates. Filaments less than 3 µm thick, abundant, and whitish.

##### Habitat.

Cave, rocky bottom, *Posidonia oceanica* meadow, coralligenous community. Bathymetric range 1–100 m.

##### Mediterranean Caves.

Blue, La Catedral, J1, Meda Petita, Petita de la Vaca, Misidacis caves (Balearic Sea); Bagaud, Endoume, Figuier, Trèmies caves (Gulf of Lions); Zoagli-Chiavari Cave (Ligurian Sea); Misteri, Gaiola, Tuffo Tuffo caves (Central Tyrrhenian Sea); Molare caves (Southern Tyrrhenian Sea); Monastir, Salakta caves (Sicily Channel); Leuca caves (Ionian Sea); Stražica Cave (Northern Adriatic Sea); Arenile, Pagliai, Viole, Coccodrillo, Cala Tonda, Bue Marino, Rondinelle, Pecore, Regina caves (Southern Adriatic Sea) (Sarà 1958, 1959, 1961a, b, 1962, 1964a, 1968; Labate 1965; Melone 1965; Rützler 1966; Pouliquen 1972; Bibiloni et al. 1984a, 1989; Corriero et al. 2000; Novosel et al. 2002; Ben Mustapha et al. 2003; Harmelin et al. 2003; Martì et al. 2004; Pronzato and Manconi 2011).

##### Remarks.

The present description is based on the holotype LMJG 15499 (Museum Joanneum of Graz, Austria), O. Schmidt collection, from Lesina (Adriatic Sea), and other specimens belonging to the Schmidt’s collection preserved in the same museum. The study in depth of this dry holotype material resulted in the evidence that it does not belong to the genus *Ircinia* but perfectly matches the genus *Sarcotragus*. The holotype is, probably, a fragment of a bigger specimen and does not exceed 15 cm in diameter; no traces of dermal membrane or choanosomal architecture are visible, suggesting that it can be a beached specimen. The type material of Pallas *Spongia fasciculata* is missing and the single specimen of *Ircinia fasciculata* belonging to the Schmidt’s collection (NHMG 15499) must be ascribed to the genus *Sarcotragus*. Pronzato et al. (2004) investigated the species formerly named *Ircinia fasciculata* (Pallas, 1766); the result was that *Ircinia variabilis* (Schmidt, 1862) became the type species of the genus *Ircinia* Nardo, 1833 and the specimen LMJG 15499, of *Ircinia fasciculata*, was moved under the genus *Sarcotragus* Schmidt, 1862 affirming that: “a further study will decide if this species is a good one or a synonym”. Pronzato et al. (2004) focused the problematic status of the taxon but did not describe the species. Here a new combination for *Sarcotragus fasciculatus* is proposed. *Sarcotragus fasciculatus* is clearly different from the other species ascribed in the genus, also when compared with extra-Mediterranean species (Pronzato et al. 2004) because all its fibres are free of inclusions and primary ones are formed by “fascicules of secondaries”.

**Figure 20:**
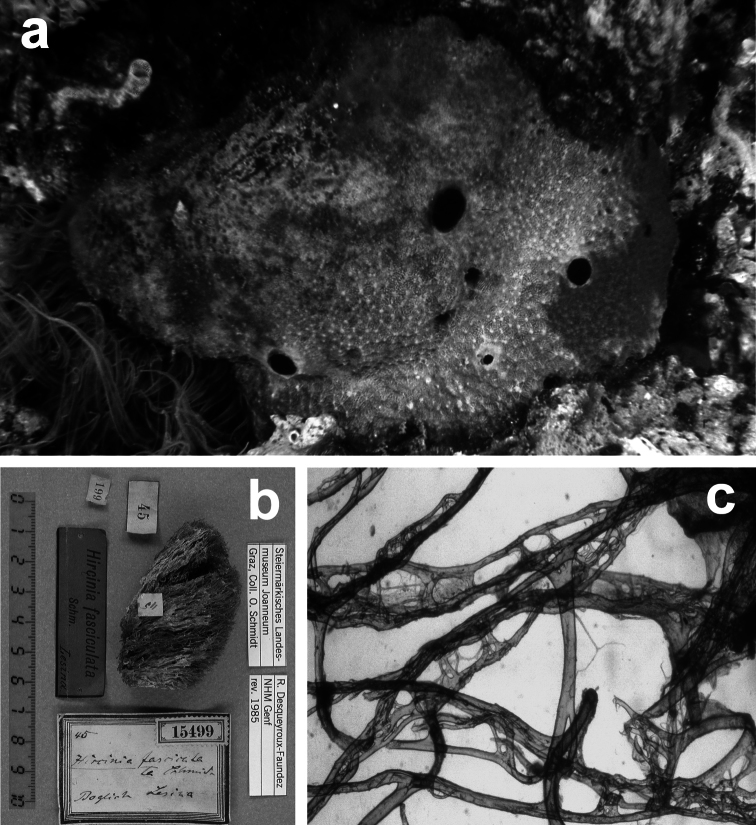
*Sarcotragus fasciculatus*. **a** living specimen (*ca*. 7 cm) **b** type specimen 15499 of the Schmidt’s collection preserved in the Landes Museum Joanneum of Graz **c** skeletal network without inclusions in primary fibres (detail of b). **b, c** modified from Pronzato *et al*. (2004).

#### Sarcotragus foetidus

Schmidt, 1862

http://species-id.net/wiki/Sarcotragus_foetidus

Fig. 21

Sarcotragus foetidus Schmidt, 1862: 36.

##### Description.

Growth form irregularly massive to globular (up to 1 m in diameter, 50 cm in height); oscules large (0.5–1 cm in diameter) with a short collar, often grouped in a central depression at the top of the body. Consistency soft and strong. Colour is medium grey, but brown or black varieties have been also recorded (Vacelet 1959). Surface is smooth or covered by several epizoans. Conules are 2–3 mm high and 10–15 mm apart. Dry specimens become very hard and smaller (1/5) than living ones, also colour changes regularly into black. The skeleton does not differ from the other Mediterranean species belonging to the genus; the main skeleton composed by a reticulate network of primary (*ca*. 100–200 µm in diameter) and secondary (*ca*. 50–100 µm in diameter) fibres. Filaments abundant (1–3 µm in diameter).

##### Habitat.

Cave, rocky, detritic and muddy bottom, coralligenous community. Bathymetric range 3–400 m.

##### Mediterranean Caves.

Blava, Calamars, Meda Petita, Petita de la Vaca caves (Balearic Sea); Mago Cave (Central Tyrrhenian Sea); Taccio Vecchio 1 Cave-Lampedusa*, Tabarka Tunnel (Sicily Channel); Croatian caves (Northern Adriatic Sea); Viole Cave (Southern Adriatic Sea); Chios 213, Trypia Spilia, Farà, Agios Vasilios caves (Aegean Sea) (Pansini et al. 1977; Bibiloni et al. 1984a; Uriz et al. 1992; Voultsiadou-Koukoura and Koukouras 1993; Ben Mustapha et al. 2002; Pronzato and Manconi 2011; Bakran-Petricioli et al. 2012; Cadeddu 2012; Gerovasileiou and Voultsiadou 2012).

**Figure 21:**
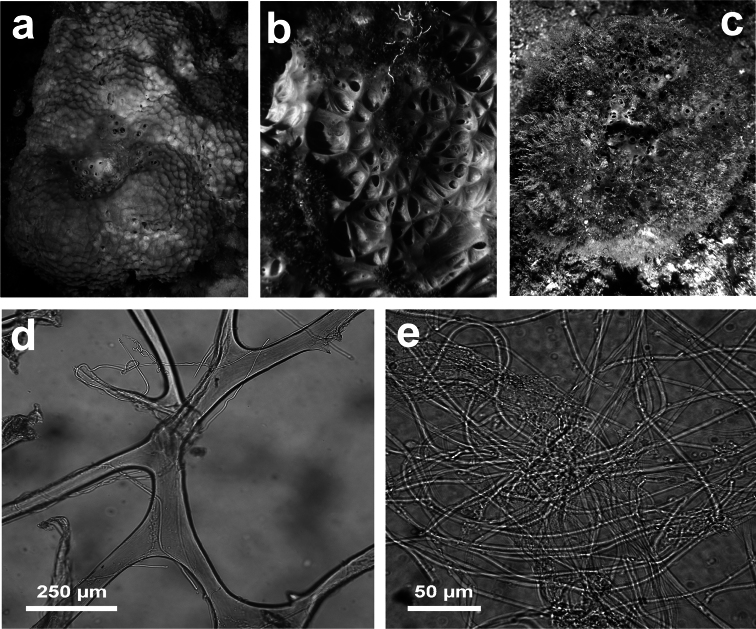
*Sarcotragus foetidus*. **a** a large (*ca*. 40 cm) living specimen free of epibiotic organisms **b** magnification of the sponge surface network **c** large specimen (*ca*. 35 cm) with dense epibiotic organisms **d** uncored skeleton fibre **e** very thin filaments.

#### Sarcotragus pipetta

(Schmidt, 1868)

http://species-id.net/wiki/Sarcotragus_pipetta

Fig. 22

Hircinia pipetta Schmidt, 1868: 5.

##### Description.

Growth form massive (10 × 10 cm to 5 × 5 cm) and irregular in the basal portion with 5 to 10 peculiar, unequal, hollow, conical processes (1 to 3 cm high and 1 to 2 cm wide at their base) bearing an apical, circular oscule 1 to 3 mm in diameter. Consistency firm and elastic, difficult to tear. Colour in formalin from light brown to dark violet-brown to rarely greyish azure *in vivo* (Mitigliano cave). Dermal membrane with fine particles of sand. Conules *ca*. 0.5 mm in height, rather irregularly distributed (1 to 3 mm apart). Skeleton reticulate with meshes 2–3 mm in diameter. Primary fibres with fasciculate architecture, with a central fibre (50 to 150 µm thick) cored by small inclusions (mainly sand) irregularly surrounded by a trellis of thinner fibres (20 to 40 µm thick), free of inclusions. These complex fibres assume here and there the shape of a perforated plate (400–700 µm in diameter). Secondary fibres simple, moderately cored by foreign matter, generally narrow at their centre and anastomosing to the main fibres by root-like processes. Filaments up to 6.5 µm in thickness.

##### Habitat.

Cave, rocky bottom, coralligenous community. Bathymetric range 8–120 m.

##### Mediterranean caves.

Mitigliano Cave (Central Tyrrhenian Sea) (Pansini and Pronzato 1982; Balduzzi et al. 1989; Pronzato and Manconi 2011).

**Figure 22:**
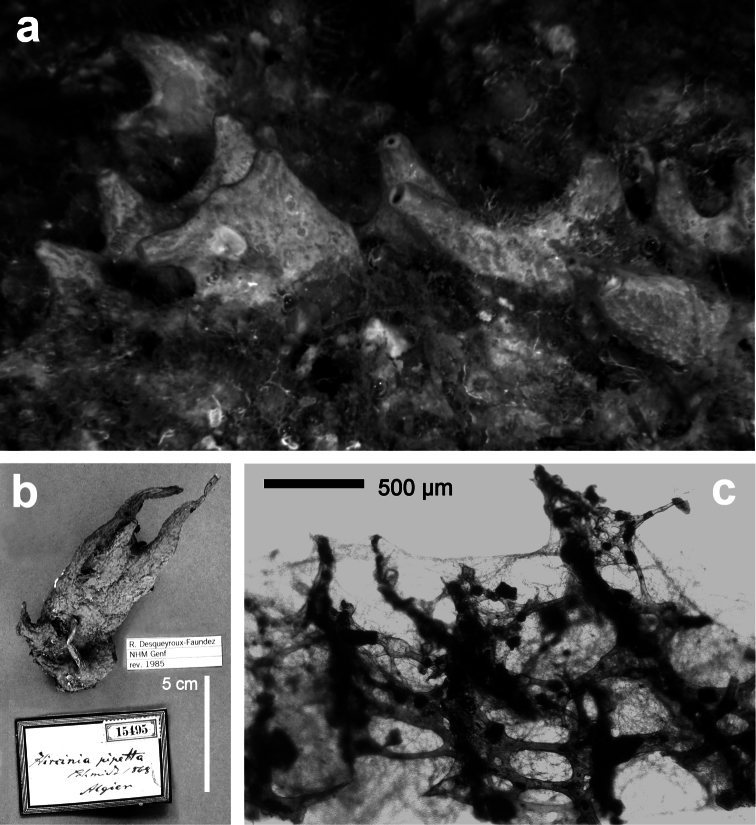
*Sarcotragus pipetta*. **a** living specimen in the Mitigliano Cave **b** type specimen 15495 from the Algerian coasts of the Schmidt’s collection in the Landes Museum Joanneum of Graz **c** skeletal network close to the sponge surface (LM) with ascending primary fibres supporting conules and filaments.

#### Sarcotragus spinosulus

(Schmidt, 1862)

http://species-id.net/wiki/Sarcotragus_spinosulus

Fig. 23

Sarcotragus spinosulus Schmidt, 1862: 35.

##### Description.

Growth form regular, massive, rarely exceeding 10 cm in diameter. Colour black or dark grey *in vivo*. Consistency strong, relatively elastic. Surface finely conulose (1–2 mm in height and 2–3 mm apart). Oscules (up to 1 cm in diameter) irregularly scattered. Skeleton network reticulation of ascending primary fibres (90–180 µm in diameter) with a fibrous narrow core free of inclusions or bearing only rare spicules. Secondary fibres (50–100 µm in diameter) uncored and laminated. Filaments (0.7–2.0 µm in diameter) very abundant giving a strong consistency.

##### Habitat.

Cave, rocky, detritic and muddy bottom, coralligenous community, lagoon, *Posidonia oceanica* meadow, epibiotic on *Pinna nobilis*. Bathymetric range 1–60 m.

##### Mediterranean caves.

Blava, La Catedral, Meda Petita, Petita de la Vaca caves (Balearic Sea); Bear, Troc, Endoume caves (Gulf of Lions); Isolotto, Mago, Tuffo Tuffo caves (Central Tyrrhenian Sea); Porto Cesareo Cave (Ionian Sea); Croatian, Stražica caves (Northern Adriatic Sea); Viole, Bue Marino, Piccolo Ciolo, Marinella, Principessa caves (Southern Adriatic Sea); Ftelio Cave (Aegean Sea) (Rützler 1966; Boury-Esnault 1971; Pouliquen 1972; Pulitzer-Finali and Pronzato 1976, 1980; Pansini et al. 1977; Pulitzer-Finali 1977; Bibiloni et al. 1984a, 1989; Corriero et al. 2000, 2004; Bussotti et al. 2006; Novosel et al. 2002; Turon et al. 2009; Pronzato and Manconi 2011; Bakran-Petricioli et al. 2012; Gerovasileiou and Voultsiadou 2012).

**Figure 23:**
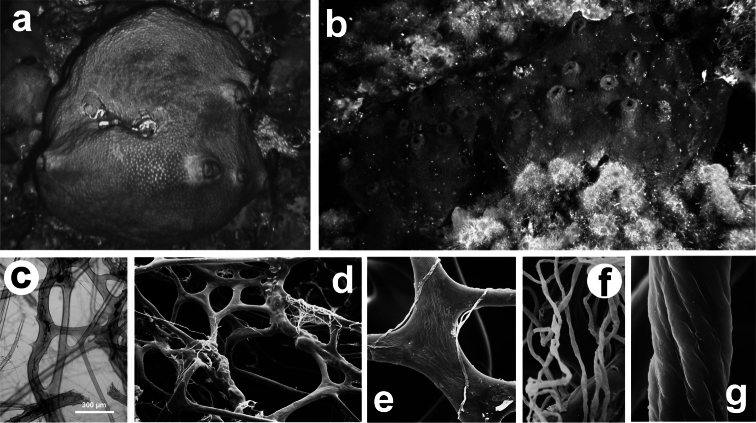
*Sarcotragus spinosulus*. **a, b** specimens with different growth form **c–g** different magnifications of skeletal network with primary and secondary fibres, and filaments (LM and SEM).

#### Coscinoderma sporadense

Voultsiadou-Koukoura, Van Soest and Koukouras, 1991

http://species-id.net/wiki/Coscinoderma_sporadense

Fig. 24

Coscinoderma sporadense Voultsiadou-Koukoura, Van Soest and Koukouras, 1991: 195.

##### Description.

Growth form massive, cushion shaped, lobose (6 to 30 cm^2^ surface area, *ca*. 5 mm avg thickness). Colour light brown, lighter in formalin. Consistency soft, spongy and compressible. Surface conulose with conules *ca*. 1 mm in height and 2–4 mm apart. Oscules few (2–4 mm in diameter). Ostia visible in some areas with a diameter of 50–200 µm. Ectosome (100–350 µm in thickness) detachable and armoured with sand grains and foreign spicules.

Ascending primary fibres (50–80 µm in diameter) cored with foreign material to such a degree that sometimes spongin is hardly visible. Foreign material usually sand grains mixed with low amounts of spicules, although some fibres cored exclusively with spicules. Primary fibres connected to a dense, irregular, network of secondary fibres which, in the vicinity of the primary fibres, has the form of a perforated plate. Secondary fibres (10–40 µm in diameter) often with rounded or broadly acute free tips, thin and hardly anastomosing. The secondary network, in its greater part, resembles an unwound clew.

##### Habitat.

Cave, rocky bottom. Bathymetric range 3–15 m.

##### Mediterranean Caves.

Youra Cave (Sporades Islands, Northern Aegean Sea) (Voultsiadou-Koukoura et al. 1991; Pronzato and Manconi 2011).

**Figure 24:**
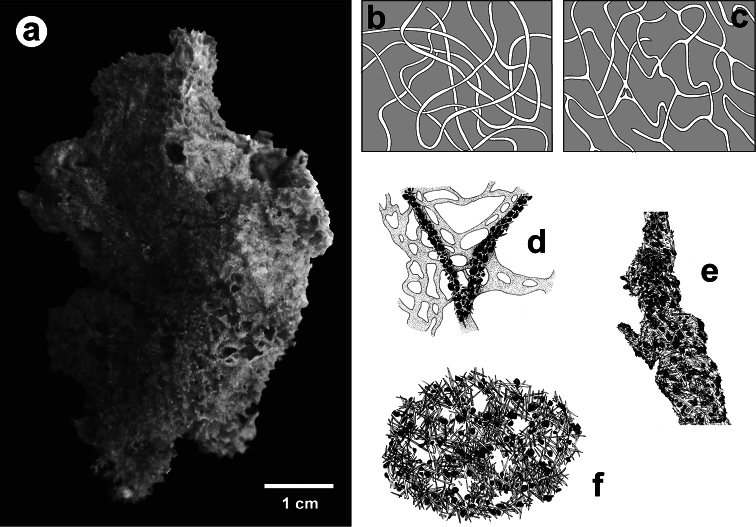
*Coscinoderma sporadense*. **a** type specimen **b, c** network architecture of almost transparent secondary fibres **d** connections between primary and secondary fibres **e** primary fibre completely cored by inclusions **f** close-up of the sponge’s surface engulfing mineral grains and spicules. **a–f** modified from Voultsiadou-Koukoura et al. (1991).

#### Hippospongia communis

(Lamarck, 1813)

http://species-id.net/wiki/Hippospongia_communis

Fig. 25

Spongia communis Lamarck, 1813: 370.

##### Description.

Growth form massive, rounded. Colour *in vivo* dark grey. Surface with large, sparse conules. Oscules scattered or grouped at the top surface, pre-oscular cavities extremely developed, large subdermal canals radially arranged at oscula. Large cavernous cavities (1–4 cm) irregularly scattered in the choanosome. Skeleton reticulate with ascending main fibres supporting the conules. Primaries (60–100 µm in diameter) twisted, with inclusions (fragments of spicules and mineral granules). Primaries present exclusively as main axis of conules, towards the surface, in some specimens/populations. Secondaries (20–30 µm in diameter) abundant, forming a dense network, without inclusions.

##### Habitat.

Cave, coralligenous community, *Posidonia oceanica* meadow, rocky/detritic/muddy bottom. Bathymetric range 1–200 m.

##### Mediterranean caves.

Blava, Blue, La Catedral caves (Balearic Sea); Endoume, Figuier, Trèmies caves (Gulf of Lions); Azzurra, Mago caves (Central Tyrrhenian Sea) (Pouliquen 1972; Pulitzer-Finali and Pronzato 1976, 1980; Cinelli et al. 1977; Pansini et al. 1977; Pulitzer-Finali 1977; Bibiloni et al. 1989; Martì et al. 2004; Turon et al. 2009; Pronzato and Manconi 2011).

**Figure 25:**
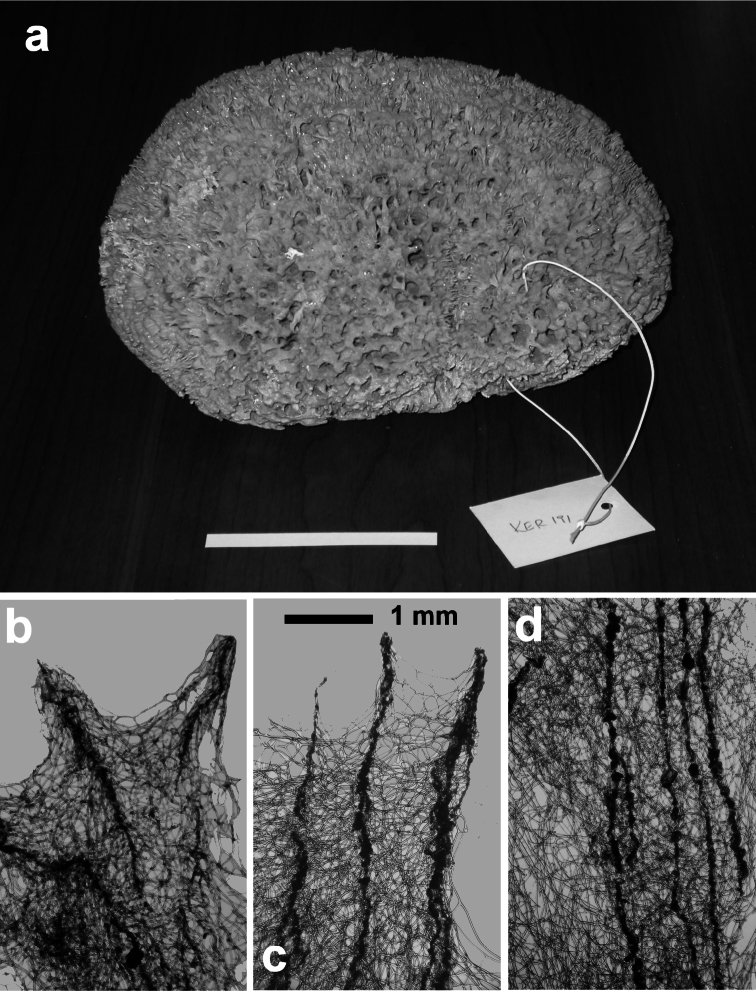
*Hippospongia communis*. **a** a large, over 25 cm, specimen collected along the Libyan coast **b, c** skeletal network with tips of primary cored fibres supporting conules at the sponge surface **d** ascending tracts of primary fibres in the choanosome.

#### Spongia lamella

(Schulze, 1879)

http://species-id.net/wiki/Spongia_lamella

Fig. 26

Euspongia officinalis lamella Schulze, 1879a: 617.

##### Description.

Growth form vase- or fan-shaped, large (up to over 1 m). Surface finely conulose, inhalant and exhalant openings of the aquiferous system on the outer and inner sides, respectively, of the vase, or on the opposite sides of the fan. Wall 5–10 mm thick. Inhalant apertures large and irregular. Oscules small with a diameter *ca*. 1.5 mm and grouped in clubs regularly scattered. Colour *in vivo* from grey to brown. Surface conulose. Ectosomal skeleton covered by a dermal membrane rich of sand, as a network of secondary fibres (15–20 µm in diameter) connected to the apices of primaries. Choanosomal skeleton as an irregular network of secondaries (20–40 µm in diameter) with evident tracts of primary fibres (50–80 µm in diameter) extended between inner and outer surfaces. Primary fibres cored by mineral inclusions.

##### Habitat.

Cave, rocky/muddy/detritic bottom. Bathymetric range from shallow water to 22–300 m.

##### Mediterranean caves.

Galatea*, Falco*, Bisbe* caves (Sardinian Sea); Trèmies Cave (Gulf of Lions); Bergeggi Cave (Ligurian Sea) (Pouliquen 1972; Bianchi and Morri 1994; Manconi et al. 2011; Pronzato and Manconi 2011; Cadeddu 2012).

**Figure 26:**
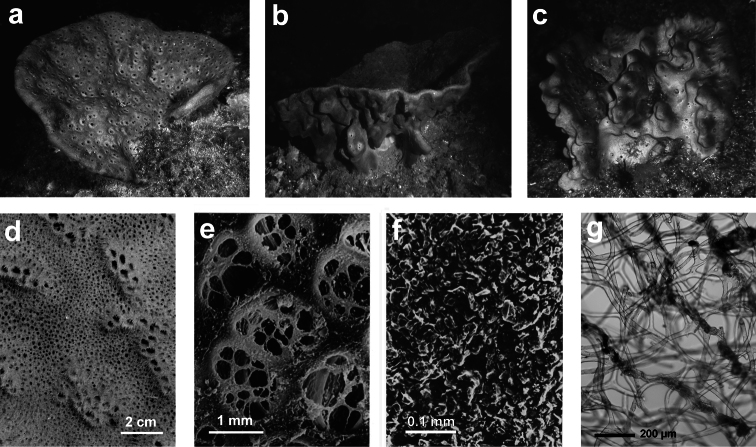
*Spongia lamella*. **a–c** different growth forms **d** grouped oscules in the inner exhalant sponge surface **e** detail (SEM) of the inhalant apertures **f** detail of sponge surface with mineral grains enclosed in the slim collagenous layer **g** skeletal network of a lamina with abundant, cored primary fibres extended between the inner and outer surfaces, and inter-connected by a network of thinner secondary fibres without inclusions.

#### Spongia nitens

(Schmidt, 1862)

http://species-id.net/wiki/Spongia_nitens

Fig. 27

Ditela nitens Schmidt, 1862: 24, 1864.

##### Description.

Growth form irregularly lobate, rarely larger than 15–20 cm. Oscules (2 mm in diameter) on each lobe, with evident very long converging exhalant canals. Consistency soft and strong. Colour whitish to light brown. Conules small and regular. Primary fibres (40–60 µm in diameter) sometimes showing a fibrous opaque core, avoiding inclusion or with rare spicule fragments. Secondary fibres (20–35 µm in thickness) connecting primary ones in a regular network; a second superficial network is formed by thinner (4–10 µm) fibres. Skeleton extremely soft. The specific name refers to the silky sponge’s surface with an external membrane smooth and translucent.

##### Habitat.

Cave, coralligenous community. Bathymetric range 0–15 m.

##### Mediterranean caves.

Falco*, Bisbe* caves (Sardinian Sea); Endoume, Figuiers caves (Gulf of Lions); Leuca caves (Ionian Sea); Croatian caves (Northern Adriatic Sea); Farà, Agios Vasilios caves (Aegean Sea)(Sarà 1968; Pouliquen 1972; Pronzato and Manconi 2011; Bakran-Petricioli et al. 2012; Cadeddu 2012; Gerovasileiou and Voultsiadou 2012).

**Figure 27:**
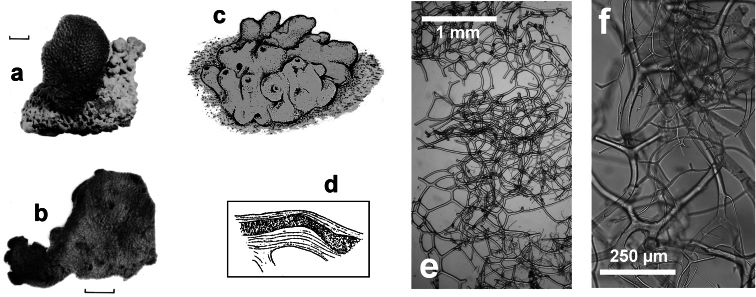
*Spongia nitens*. **a, b** dry specimens of the Schmidt’s collection preserved in the Landes Museum Joanneum of Graz **c** drawing of a living specimen **d** fibre showing an opaque narrow core **e, f** different magnification (LM) of the skeletal network, entirely free of mineral inclusions. **a, b** modified from Desqueyroux-Faundez and Stone (1992) **c, d** modified from Vacelet (1987) **a, b** scale bars = 1 cm.

#### Spongia officinalis

Linnaeus, 1759

http://species-id.net/wiki/Spongia_officinalis

Fig. 28

Spongia officinalis Linnaeus, 1759: 1348 (*partim*).

##### Description.

Growth form massive-lobate, surface finely conulose, single oscules scattered or at the apex of lobes, pre-oscular cavities well evident. Colour *in vivo* from light grey to black. Ectosomal skeleton as apices of primary fibres joining secondary fibres to form the conical reticulum which supports the conules. Choanosomal skeleton: network dense with irregular polygonal meshes of secondaries joining to form ascending primaries. Primary fibres (50–100 µm in diameter) typically twisted with ornamentations as parallel ridges along the main fibre axis mainly developed and evident towards the surface, cored with sand grains and spicules. Secondaries (20–35 µm in diameter) with ornamentations as parallel ridges along the main fibre axis, twisted and characterised by concentric layers of compact spongin surrounding the compact axial core without inclusions.

##### Habitat.

Cave, coralligenous community, rocky/detritic/muddy/sandy bottom, lagoon, coralligenous community, *Posidonia oceanica* meadow. Bathymetric range 1–70 m.

##### Mediterranean caves.

Meda Petita, Petita de la Vaca caves (Balearic Sea); Falco*, Bisbe* caves (Sardinian Sea); Endoume, Figuiers, Trèmies, Niolon, Bagaud caves (Gulf of Lions); Bergeggi, Eastern-Bonassola, Zoagli-Chiavari caves (Ligurian Sea); Azzurra, Isolotto, Mago, Misteri, Tuffo Tuffo caves (Central Tyrrhenian Sea); Taccio Vecchio 1 Cave-Lampedusa*, Cani Islands Tunnel (Sicily Channel); Leuca caves (Ionian Sea); Croatian, Vrbnik-Krk caves (Northern Adriatic Sea); Pagliai, Regina caves (Southern Adriatic Sea) (Laborel and Vacelet 1958; Sarà 1959, 1964a; Vacelet 1959; Labate 1965; Rützler 1966; Pouliquen 1972; Pulitzer-Finali and Pronzato 1976, 1980; Cinelli et al. 1977; Pansini et al. 1977; Pulitzer-Finali 1977; Bibiloni et al. 1984a, b; Bianchi et al. 1986; Arko-Pjevac et al. 2001; Ben Mustapha et al. 2002; Harmelin et al. 2003; Manconi et al. 2011; Pronzato and Manconi 2011; Bakran-Petricioli et al. 2012; Cadeddu 2012).

**Figure 28:**
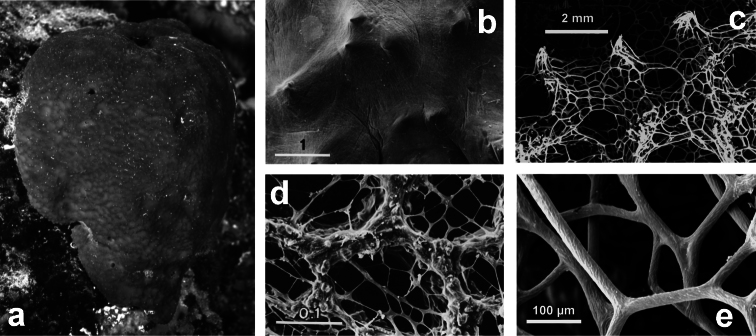
*Spongia officinalis*. **a** massive large living specimen (*ca*. 25 cm) showing a finely conulose surface with scattered small oscula **b** close up of the conulose surface covered by a thin uncellularized collagenous layer (SEM) **c** magnifications of an inhalant cribrose basal area (SEM) **d** conules at the spongin skeleton surface (SEM) **e** twisted surface of secondary fibres (SEM). **b, c** modified from Pronzato et al. (1998) **d, e** modified from Pronzato & Manconi (2008) **b, d** scale bars in mm.

#### Spongia virgultosa

(Schmidt, 1868)

http://species-id.net/wiki/Spongia_virgultosa

Fig. 29

Euspongia virgultosa Schmidt, 1868: 4.

##### Description.

Growth form encrusting (*ca*. 2–5 cm in diameter), rarely massive (up to 10–15 cm), usually emerging from the substratum only with inhalant and exhalant funnels (5–15 mm high, 3–5 mm in diameter). Sponge surface irregularly conulose (1–2 mm high, 24 mm apart). Colour from light to very dark brown. Primary fibres (40–50 µm) cored by mineral debris, extremely rare and often absent; secondaries extremely variable (10–50 µm).

##### Habitat.

Cave, coralligenous community, detritic/muddy bottom, lagoon, artificial reef, *Posidonia oceanica* meadow, epibiotic on *Pinna nobilis*. Generally covered by epibionts in turbulent superficial water. Bathymetric range 1–50 m.

##### Mediterranean caves.

La Catedral, J2, Blue, Meda Petita, Petita de la Vaca, Misidacis caves (Balearic Sea); Galatea*, Falco*, Bisbe* caves (Sardinian Sea); Bear, Troc, Endoume, Figuiers, Trèmies caves (Gulf of Lions); Punta Carega, Manara, Zoagli-Chiavari caves (Ligurian Sea); Azzurra, Isolotto, Mago, Lacco Ameno, Misteri, Gaiola, Tuffo Tuffo, Mitigliano caves (Central Tyrrhenian Sea); Porto Cesareo Cave (Ionian Sea); Croatian caves (Northern Adriatic Sea); Pagliai, Viole, Pecore, Arenile, Coccodrillo, Rondinelle, Bue Marino, Piccolo Ciolo, Marinella, Regina caves (Southern Adriatic Sea); Trypia Spilia, Farà, Ftelio caves (Aegean Sea) (Sarà 1960a, b, 1961a, 1964a; Labate 1965; Rützler 1966; Boury-Esnault 1971; Pouliquen 1972; Pansini et al. 1977; Pulitzer-Finali and Pronzato 1980; Pansini and Pronzato 1982; Bibiloni et al. 1984a, 1989; Balduzzi et al. 1989; Corriero et al. 2000, 2004; Martì et al. 2004; Pronzato and Manconi 2011; Bakran-Petricioli et al. 2012; Cadeddu 2012; Gerovasileiou and Voultsiadou 2012).

**Figure 29:**
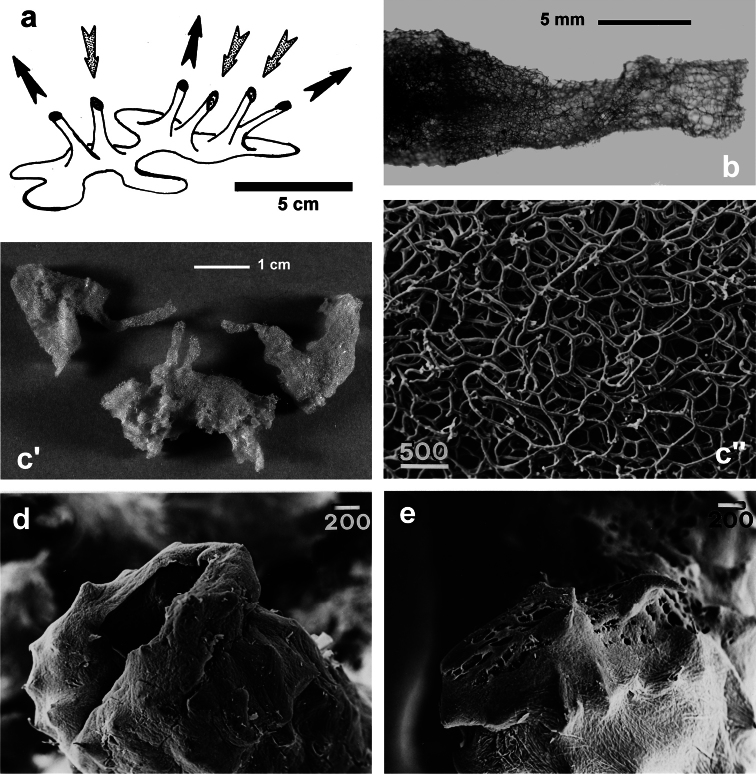
*Spongia virgultosa*. **a** schematic drawing of the aquiferous system architecture and direction of incurrent and excurrent water flow **b** low magnification of the skeleton (LM) supporting a funnel **c'** spongin skeletons of some specimens showing the exhalant funnels (arrows) of the aquiferous system **c''** blowup of skeleton skeleton characterised by the absence of cored primary fibres (LM) **d** exhalant funnel (SEM) **e** inhalant funnel (SEM). c-e) modified from Pronzato et al. (1998). **d, e, f** scale bars in µm.

#### Spongia zimocca

Schmidt, 1862

http://species-id.net/wiki/Spongia_zimocca

Fig. 30

Spongia zimocca Schmidt, 1862: 23.

##### Description.

Massive to globular growth form, small size, usually not over 15 cm in diameter. Surface softly hairy, densely conulose with very long conules (2–3 mm high and less than 1 mm apart) sometimes a single conule supported by 2–3 converging primary fibres. Oscules not evident and located in small deep superficial depressions. Colour *in vivo* never reported. Consistency very soft, elastic and strong. Skeleton as a network of regular meshes (100–200 µm) with primary fibres bearing very rare inclusions (particularly fragments of spicules) and secondaries completely free of inclusions; primary fibres typically formed by anastomosing secondaries in fascicules (50–80 µm in diameter).

##### Habitat.

Cave, rocky bottom, coralligenous community. Bathymetric range 1–40 m. Here we report a new record from the Bisbe Cave in the NW-Sardinian karst.

##### Mediterranean caves.

Bisbe* Cave (Sardinian Sea); Salakta Caves (Sicily Channel) (Ben Mustapha et al. 2003; Manconi et al. 2011; Pronzato and Manconi 2011; Cadeddu 2012).

##### Remarks.

It is a problematic species, indeed the Schmidt’s type specimen (naked skeleton, Cyprus, no further data), preserved in the Graz Museum (LMJG 15470/0) is clearly a *Spongia officinalis*. Moreover many authors, in various papers, described this species differently, contributing to determine its problematic taxonomic status. In contrast with that, the commercial “Zimoccas” really belong to a species distinctly different from the other specieshitherto ascribed tothe genus *Spongia* as reported also by Schmidt (1862), Schulze (1879a) and de Laubenfels (1948). As a consequence the Graz Museum type needs to be carefully studied. The present description is based on the specimens TRG Ker 346, DTRG Ker 347, Jerba-El-Jem (Tunisia), 3–4 m, soft bottom, August 2006. Many traders consider “Zimocca” as the best commercial Mediterranean sponge.

**Figure 30:**
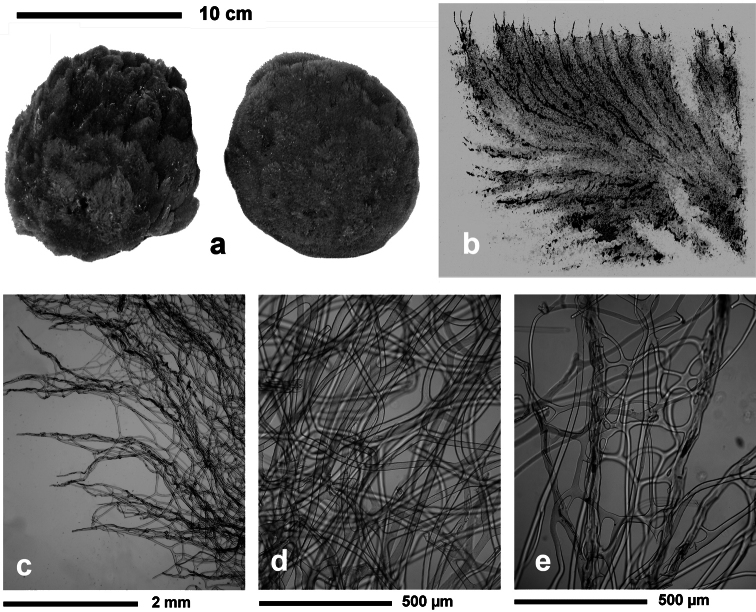
*Spongia zimocca*. **a** specimens from the sponge market (Djerba, Tunisia) **b** drawing of the skeletal network at the sponge surface **c** long and dense conules supported by tips of primary fibres at the sponge surface (LM) **d** network of uncored secondary fibres **e** cored primary fibres among uncored secondaries. **b** modified from Schulze (1879a).

#### Cacospongia mollior

Schmidt, 1862

http://species-id.net/wiki/Cacospongia_mollior

Fig. 31

Cacospongia mollior Schmidt, 1862: 27.

##### Description.

Growth form massive, lobate, 10–25 cm in diameter. Consistency soft and spongy, easy to tear off *in vivo* and friable when dry. Colour dark grey with whitish, bluish and magenta tinges. Surface smooth, regularly conulose (1–1.5 mm in height, 1–2 mm apart), forming regular characteristic “circular craters”. Oscules scattered, small and single, upwards of 1 mm in diameter. Flagellate chambers spherical, 30–45 µm in diameter. Skeleton network reticulate with regular meshes (300–600 µm). Primary ascending fibres (80–120 µm) cored by mineral debris; secondaries abundant, free of inclusions, transparent and uncored. Skeleton soft when hydrated and brittle when dry.

##### Habitat.

Cave, coralligenous community, rocky/detritic/muddy bottom, *Posidonia oceanica* meadow, lagoon, epibiotic on *Pinna nobilis*. Bathymetric range 1–100 m.

##### Mediterranean caves.

Blava, Calamars, Misidacis caves (Balearic Sea); Bear, Endoume, Figuiers, Trèmies, Bagaud caves (Gulf of Lions); Azzurra, Mago caves (Central Tyrrhenian Sea); Bue Marino Cave (Southern Adriatic Sea); Ftelio Cave (Aegean Sea) (Boury-Esnault 1971; Pouliquen 1972; Pulitzer-Finali and Pronzato 1976, 1980; Pansini et al. 1977; Pulitzer-Finali 1977; Uriz et al. 1992; Corriero et al. 2000; Harmelin et al. 2003; Martì et al. 2004; Pronzato and Manconi 2011; Gerovasileiou and Voultsiadou 2012).

**Figure 31:**
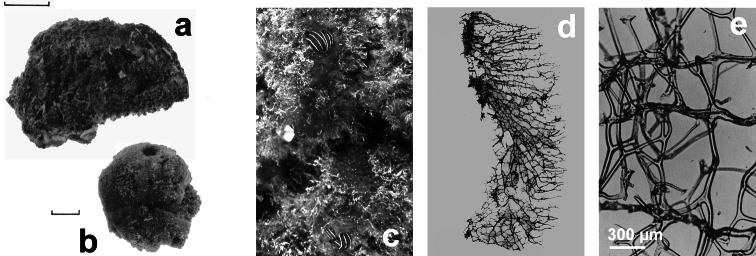
*Cacospongia mollior*. **a, b** dry specimens from the Schmidt’s collection preserved in the Landes Museum Joanneum of Graz **c** close up of the sponge surface harbouring several specimens of *Chromodoris* spp. grazing on epibionts **d** skeletal network with primary (cored) and secondary (uncored) fibres close to the sponge surface **e** close up of the skeletal network with primary and secondary fibres (LM). **a, b** modified from Desqueyroux-Faundez and Stone (1992) **d** modified from Schulze (1879a) **e** modified from Pulitzer-Finali & Pronzato (1976) **a, b** scale bars = 1 cm.

#### Cacospongia proficens

Pulitzer-Finali and Pronzato, 1980

http://species-id.net/wiki/Cacospongia_proficens

Fig. 32

Cacospongia proficens Pulitzer-Finali and Pronzato, 1980: 141.

##### Description.

Growth form massive at the basal portion with several ascending conical processes each bearing a small apical oscule. Specimen designated as the holotype, measures 6 × 7 cm at the base, and has about ten processes up to 2 cm high, 12–13 mm wide at their base. Consistency soft and easy to tear. Colour in formalin grey, cream internally. Surface conulose with no sand in the dermal membrane. Conules sharp, *ca*. 0.5 mm high and 1 mm apart. Skeleton network reticulate, irregular, with meshes 200–1100 µm wide, resembling that of *Cacospongia mollior*. Primary fibres of laminar spongin, branching, not fasciculate (50–100 µm in diameter), tapering (15–20 µm) towards the conule; they contain abundant foreign material consisting mainly of the mostly entire spicules of the associated species of *Haliclona* (*Reniera*). Secondary fibres (25–80 µm in thickness) of laminar spongin, free from inclusions.

##### Habitat.

Cave. Bathymetric range 2–15 m.

##### Mediterranean Caves.

Galatea* Cave (Sardinian Sea); Pagliai, Viole, Cala Sorrentino, Torre Incine caves (Southern Adriatic Sea) (Pulitzer-Finali and Pronzato 1980; Pronzato and Manconi 2011; Cadeddu 2012).

##### Remarks.

See remarks in *Cacospongia scalaris*.

**Figure 32:**
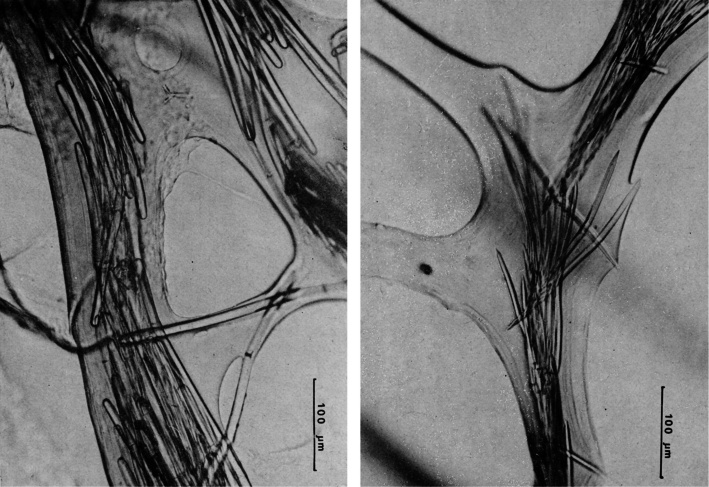
*Cacospongia proficens*. Spongin skeleton with primary fibres cored by alloctonous spicules of *Reniera cratera* (left) and *Reniera mucosa* (right). Modified from Pulitzer-Finali and Pronzato (1980).

#### Cacospongia scalaris

Schmidt, 1862

http://species-id.net/wiki/Cacospongia_scalaris

Fig. 33

Cacospongia scalaris Schmidt, 1862: 27.

##### Description.

Growth form massive, globose, lobate, large (up to 20–30 cm in diameter). Colour constantly dark grey with bluish shades. Surface conulose (conules 1–2 mm high, 2–4 mm apart) with smooth scattered circular depressions; supported by tips of primary fibres. Oscules surrounded by a short collar (up to 1 cm in diameter) abundant and irregularly scattered on the sponge’s upper part. Skeleton network lax with hard, not elastic spongin fibres. Primary fibres almost parallel, interconnected by quite perpendicular secondary fibres looking like rungs in a scale (this peculiar character originated the specific name); primary fibres (90–200 µm in diameter) cored by abundant inclusions; secondary fibres (30–80 µm in diameter) laminated with an evident fibrous core. Flagellate chambers of 30–45 µm in diameter.

##### Habitat.

Cave, rocky/detritic/muddy bottom, coralligenous community, *Posidonia oceanica* meadow, lagoon, artificial reefs, epibiotic on *Pinna nobilis*. Often on the sponge surface it is possible to find specimens ofthe nudibranch *Hypselodoris fontandraui* (Pruvot-Fol, 1951) actively grazing. Bathymetric range 1–250 m.

##### Mediterranean caves.

J1 Cave (Balearic Sea); Bear, Troc, Endoume, Figuiers, Trèmies, Niolon, Carrieres caves (Gulf of Lions); Eastern-Bonassola, Piccola Zoagli-Chiavari caves (Ligurian Sea); Mago, Secca delle Formiche-Vivara, Gaiola caves (Central Tyrrhenian Sea); Porto Cesareo Cave (Ionian Sea); Croatian, Columbera, Stražica caves (Northern Adriatic Sea); Arenile, Coccodrillo, Bue Marino caves (Southern Adriatic Sea) (Laborel and Vacelet 1958; Vacelet 1959, 1976; Sarà 1961ab, 1964a; Boury-Esnault 1971; Pouliquen 1972; Pulitzer-Finali and Pronzato 1976; Pansini et al. 1977; Pulitzer-Finali 1977; Bibiloni et al. 1989; Corriero et al. 2000, 2004; Novosel et al. 2002; Faresi et al. 2006; Pronzato and Manconi 2011; Bakran-Petricioli et al. 2012).

##### Remarks.

We do not accept that *Cacospongia scalaris* and *Cacospongia proficens* belong to the genus *Scalarispongia* on the basis of the genus diagnosis by Cook and Bergquist (2002). Indeed the comparative analysis of diagnostic traits of *Scalarispongia* vs. *Cacospongia* Schmidt, 1862 clearly indicates that no diverging morphological characters exist among them except for the ladder-like arrangement of skeletal polygonal meshes that in some species, *i.e*. *Cacospongia scalaris*, are mostly but not always rectangular. Rectangular meshes are displayed less frequently also in other species of Mediterranean cacospongias. We consider the trait ‘skeleton ladder-like with rectangular meshes’ not diagnostic at the genus level in agreement with Schmidt (1862), Vacelet (1959), Pulitzer-Finali and Pronzato (1976) and Pronzato and Manconi (2011). Moreover molecular data (see Borchiellini et al. 2004) indicate that C. *scalaris* belongs to the genus *Cacospongia*. *Cacospongia proficens* and *Cacospongia scalaris* belong therefore to the genus *Cacospongia*.

**Figure 33:**
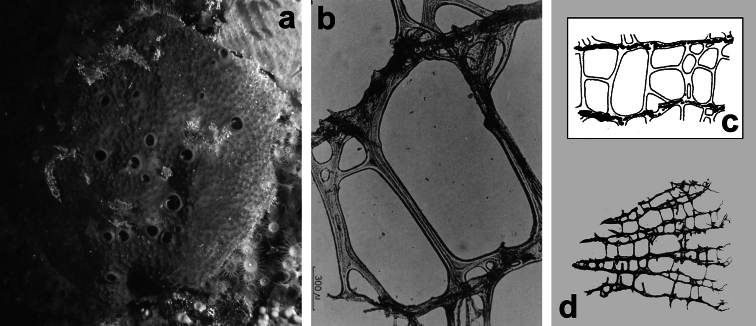
*Cacospongia scalaris*. **a** large massive specimen (*ca*. 35 cm) with finely conulose surface and evident scattered oscula **b** cored primary fibres perpendicularly connected by secondaries showing a marrow (LM) **c** drawing of the skeletal network; d) drawing showing radiating primary fibres typically connected by secondaries at right angle (90°). **b** modified from Pulitzer-Finali & Pronzato (1976) **c** modified from Laubenfels (1948) **d** modified from Schulze (1879a).

#### Fasciospongia cavernosa

(Schmidt, 1862)

http://species-id.net/wiki/Fasciospongia_cavernosa

Fig. 34

Cacospongia cavernosa Schmidt, 1862: 28.

##### Description.

Growth form tubular, massive, rounded, usually not larger than 10 cm, sometimes up to 25 cm in diameter. Colour dark brown at the surface, light yellowish at the choanosome. Large and abundant irregular cavities and canals scattered in the mesohyl (etymology of the specific name). Consistency strong and cartilaginous; sponge surface covered by very abundant conules (3–4 mm in height) giving a spiny aspect. External membrane smooth, translucent and resistant; flagellate chambers round (25–30 µm in diameter). Skeleton network very strong with large (50–250 µm) rugose or granulated fibres; some of the largest ones cored by foreign debris can be considered as primary fibres.

##### Habitat.

Cave, coralligenous community, rocky/detritic/muddy bottom, *Posidonia oceanica* meadow. Sometimes it presents a burrowing behaviour. Bathymetric range 1–367 m.

##### Mediterranean caves.

Galatea* Cave (Sardinian Sea); Bear, Endoume caves (Gulf of Lions); Giannutri Cave (Central Tyrrhenian Sea); Gozo Cave (Sicily Channel); Porto Cesareo Cave (Ionian Sea); Croatian caves (Northern Adriatic Sea); Arenile, Coccodrillo, Cala Sorrentino caves (Southern Adriatic Sea); Trypia Spilia, Madhes, Andros caves (Aegean Sea) (Boury-Esnault 1971; Pouliquen 1972; Pulitzer-Finali and Pronzato 1980; Voultsiadou-Koukoura and Koukouras 1993; Borg et al. 2004; Corriero et al. 2004; Pronzato and Manconi 2011; Bakran-Petricioli et al. 2012; Cadeddu 2012).

**Figure 34:**
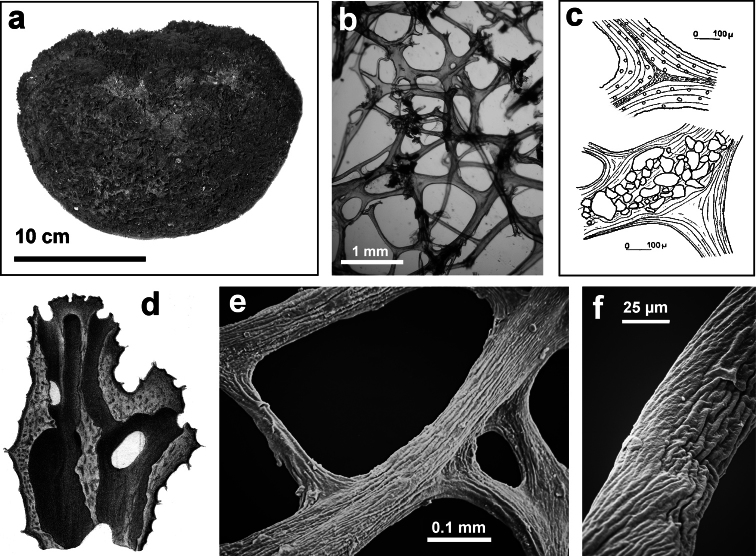
*Fasciospongia cavernosa*. **a** large specimen (over 20 cm) from the Kerkennah Islands (Tunisia) **b** stout spongin fibres in the skeletal network with very scarce inclusions at different magnifications (LM) **c** granulated (top) and cored (bottom) fibres **d** internal shape of the typical hollow (from which the species name) **e, f** rugose surface of skeletal fibres (SEM). **c** modified from Vacelet (1959) **d** modified from Schulze (1879a).

#### Hyrtios collectrix

(Schulze, 1879)

http://species-id.net/wiki/Hyrtios_collectrix

Fig. 35

Oligoceras collectrix Schulze, 1879b: 34.

##### Description.

Growth form sub-spherical or cake shaped, usually less than 10 cm in diameter. Colour black at the surface, greyish-yellow in the choanosome. Consistency very spongy in vivo, quite brittle in dry conditions. Surface conulose (conules 1–2 mm high, 1–2 mm apart). Oscules small, scattered and inconspicuous. Ectosome leathery, densely packed with highly heterogeneous detritus in nature, shape and size. Choanosome moderately cavernous and fleshy, with a ground-work of fibro-reticulations. Flagellate chambers rounded, 25–40 µm in diameter. Skeleton composed by very rare fibres completely filled by foreign materials, ascending primaries (100–350 µm in diameter), secondaries 50–100 µm, meshes very irregular in size, shape and outline; a large amount of variously composed and sized detritus is scattered in disorder in the mesohyl.

##### Habitat.

Cave, rocky/detritic bottom, coralligenous community, *Posidonia oceanica* meadow, lagoon. Bathymetric range 1–123 m.

##### Mediterranean caves.

Blava, Calamars caves (Balearic Sea); Farà Cave (Aegean Sea) (Uriz et al. 1992; Pronzato and Manconi 2011; Gerovasileiou and Voultsiadou 2012).

**Figure 35:**
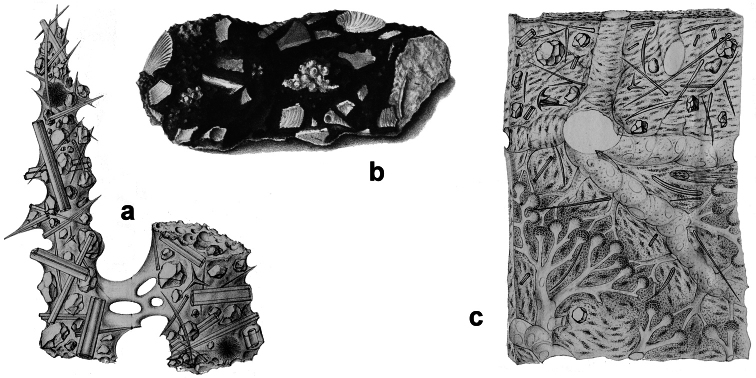
*Hyrtios collectrix*. **a** detail of a fibre tract showing a scanty amount of spongin with a wide variety of mineral debris embedded, including also spicules of many other sponge species **b** foreign materials embedded in the sponge surface **c** pictorial representation of a sponge cross section close to the surface with flagellate chambers represented as terminations of a tree-shaped aquiferous system. **a–c** modified from Schulze (1879b).

#### Halisarca dujardini

Johnston, 1842

http://species-id.net/wiki/Halisarca_dujardini

Fig. 36

Halisarca dujardini Johnston, 1842: 192.

##### Description.

Growth form encrusting, few mm thick and few cm in diameter. Consistency jelly-like or softly colloidal. Surface smooth with small oscular tubes and not evident inhalant apertures. Colour *in vivo* pale yellow to dark yellowish, sometimes with more or less dark blue tonalities. Absence of horny skeleton. Flagellate chambers radially arranged around the aquiferous system canals, elongated and typical of the genus (25 µm in diameter, 60–150 µm in length).

##### Habitat.

Cave, *Posidonia oceanica* meadow, coralligenous community, rocky/sandy bottom, frequently epibiotic on rhodophyte algae, *Ircinia* spp. and *Smittina cervicornis* (Pallas, 1766). Bathymetric range 5–100 m.

##### Mediterranean Caves.

Blava, Calamars, La Catedral, J 1, Meda petita, Petita de la Vaca caves (Balearic Sea); Troc, Bagaud caves (Gulf of Lions); Bergeggi Cave (Ligurian Sea); Secca delle Formiche-Vivara, Gaiola caves (Central Tyrrhenian Sea) (Sarà 1961a; Boury-Esnault 1971; Pulitzer-Finali and Pronzato 1976; Pulitzer-Finali 1977; Bianchi et al. 1986; Bibiloni et al. 1989; Uriz et al. 1992; Harmelin et al. 2003; Pronzato and Manconi 2011).

**Figure 36:**
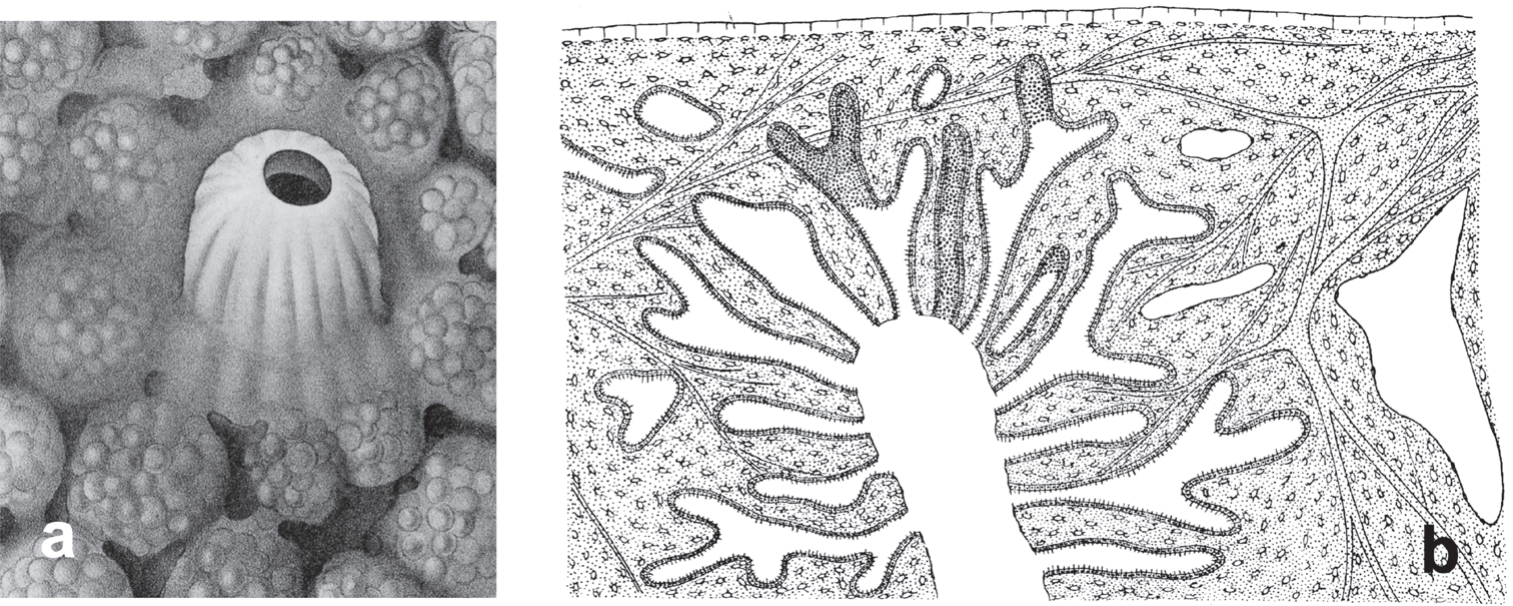
*Halisarca dujardini*. **a** drawing of the sponge surface with an osculum **b** the typical architecture of the aquiferous system. **a** modified from Schulze (1877) **b** modified from von Lendenfeld (1889).

#### Aplysina aerophoba

(Nardo, 1833)

http://species-id.net/wiki/Aplysina_aerophoba

Fig. 37

Aplysia aerophoba Nardo, 1833: 519 (preoccupied). *Aplysina aerophoba* Nardo, 1834: 714.

##### Description.

Body irregularly massive to digitate (up to 20–30 cm in diameter and height). Colour bright yellow *in vivo* and dramatically changing in a few minutes after collection or preservation (both alcohol and formalin, but also in dry conditions) into a very dark violet or most frequently pure black. Evident oscules on the top of sponge body or digitations. Sponge body surfaces seasonally covered by thin outgrowths (asexual propagules) up to 5 cm in length and 1 cm in diameter; outgrowths are lost by the mother-sponge as propagules at the end of summer. Consistency firm and fleshy. Surface smooth to slightly conulose, showing a fine (but evident) superficial fibrous network. Skeleton fragile, with fibres of a single dimensional class (80–150 µm) arranged in a regular three-dimensional scaffold. Fibre structure laminar with a large axial core (30–70 µm) inconspicuous in dry condition.

##### Habitat.

Cave, rocky/detritic/muddy bottom, lagoon, coralligenous community, *Posidonia oceanica* meadow. Bathymetric range from 10 cm to 100 m.

##### Mediterranean caves.

Meda Petita, Petita de la Vaca caves (Balearic Sea); Azzurra Cave (Central Tyrrhenian Sea); Croatian, Vrbnik-Krk, Stražica, Columbera caves (Northern Adriatic Sea); Agios Vasilios Cave (Aegean Sea) (Pulitzer-Finali and Pronzato 1980; Bibiloni et al. 1984a; Arko-Pjevac et al. 2001; Novosel et al. 2002; Faresi et al. 2006; Pronzato and Manconi 2011; Gerovasileiou and Voultsiadou 2012).

**Figure 37.**
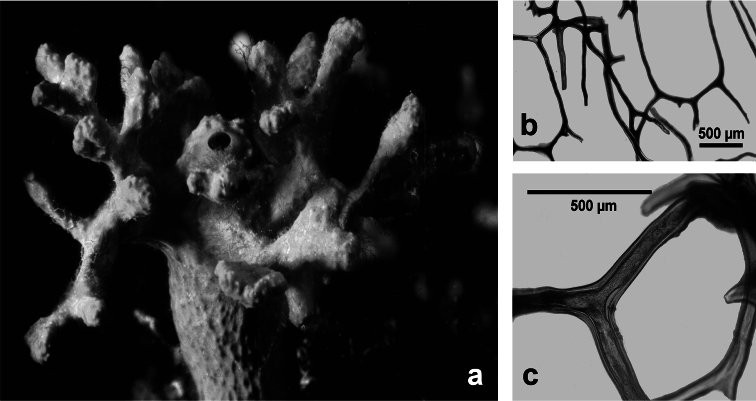
*Aplysina aerophoba*. **a**) underwater shot of a specimen with typical seasonal outgrowths in spring-summer **b, c** skeletal network at different magnifications (LM) with indistinguishable primary and secondary fibres both characterised by an empty core.

#### Aplysina cavernicola

(Vacelet, 1959)

http://species-id.net/wiki/Aplysina_cavernicola

Fig. 38

Verongia cavernicola Vacelet, 1959: 88.

##### Description.

Body shape constantly digitate (1–2 cm in diameter and 5–10 cm in height); each digitation bearing one oscule (1–3 mm) at the center of an evident apical depression. Digitations regularly arranged on a basal encrusting plate attending over 50 cm in diameter. Thin outgrowths extremely rare. Colour yellow, a little bit paler than that of *Aplysina aerophoba*. Colour tone changes after death, to medium violet in preserved specimens, never reaching very dark or black tonalities.

##### Habitat.

Cave, coralligenous community, rocky/detritic bottom. Typically sciophilous. Bathymetric range 1–110 m.

##### Mediterranean Caves.

Blava, Calamars, Meda Petita, Petita de la Vaca, Misidacis caves (Balearic Sea); Bear, Troc, Figuier, Trèmies, Bagaud caves (Gulf of Lions); Gallinara, Bergeggi, Tinetto caves (Ligurian Sea); Bonifacio, Tuffo Tuffo caves (Central Tyrrhenian Sea); Croatian, Vrbnik-Krk, Stražica, Columbera caves (Northern Adriatic Sea); Pagliai (Southern Adriatic Sea) (Vacelet 1961b; Rützler 1966; Boury-Esnault 1971; Pouliquen 1972; Bibiloni et al. 1984b; Uriz et al. 1992; Bianchi and Morri 1994; Arko-Pjevac et al. 2001; Novosel et al. 2002; Harmelin et al. 2003; Faresi et al. 2006; Tunesi et al. 2008; Pronzato and Manconi 2011; Bakran-Petricioli et al. 2012).

**Figure 38.**
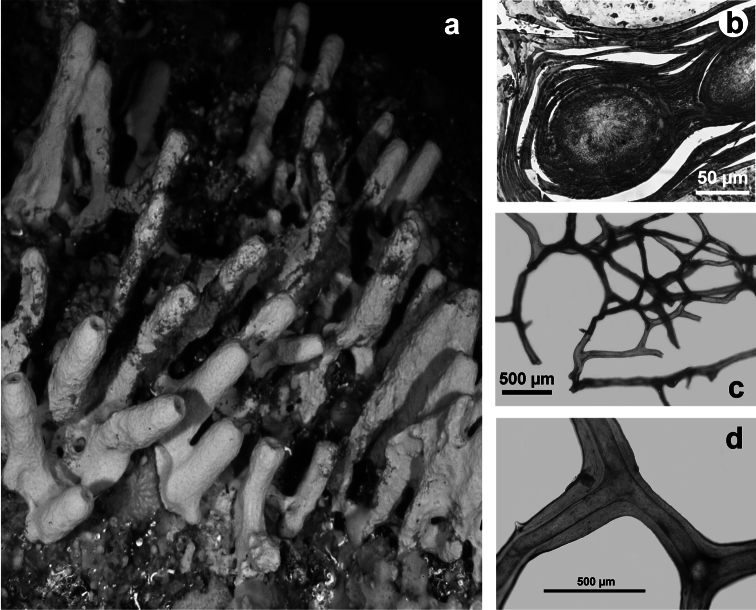
*Aplysina cavernicola*. **a** large digitate colony *ca*. 70–80 cm **b** cross section (LM) of a laminate fibre showing a light spongy core that, in dried conditions, becomes empty **c, d** different magnifications (LM) of the skeleton, indistinguishable from that of *Aplysina aerophoba*.

#### Hexadella crypta

Reveillaud, Allewaert, Pérez, Vacelet, Banaigs and Vanreusel, 2012

http://species-id.net/wiki/Hexadella_crypta

Fig. 39

Hexadella crypta Reveillaud, Allewaert, Pérez, Vacelet, Banaigs and Vanreusel, 2012: 238.

##### Description.

Growth form encrusting, cushion-like without lobes, small size, thicker than that of *Hexadella pruvoti*. Colour bright yellow to paler *in vivo*, dark purple in ethanol after releasing a purple fluid. Surface entirely striated by irregularly crossing collagenous reinforcements with some scattered, pointed conules; inconspicuous inhalant apertures and rare oscules. Ectosome rigid with collagen fibrils, nondetachable from the choanosome. Choanosome lacunar with large clusters of spherulous cells bearing large inclusions of microgranules and microgranular cells. Choanocyte chambers eurypylous, sac-shaped (ca. 30 × 20 µm in diameter). Bacteria (one type only) in the mesohyl. Aerophobins 1, 2 and isofistularin compounds with medium-high natural toxicity.

##### Habitat.

Cave. Bathymetric range 10 m.

##### Mediterranean caves.

Corail Cave (Gulf of Lions) (Reveillaud et al. 2012).

##### Remarks.

See the original description for more details and figures (Reveillaud et al. 2012).

**Figure 39.**
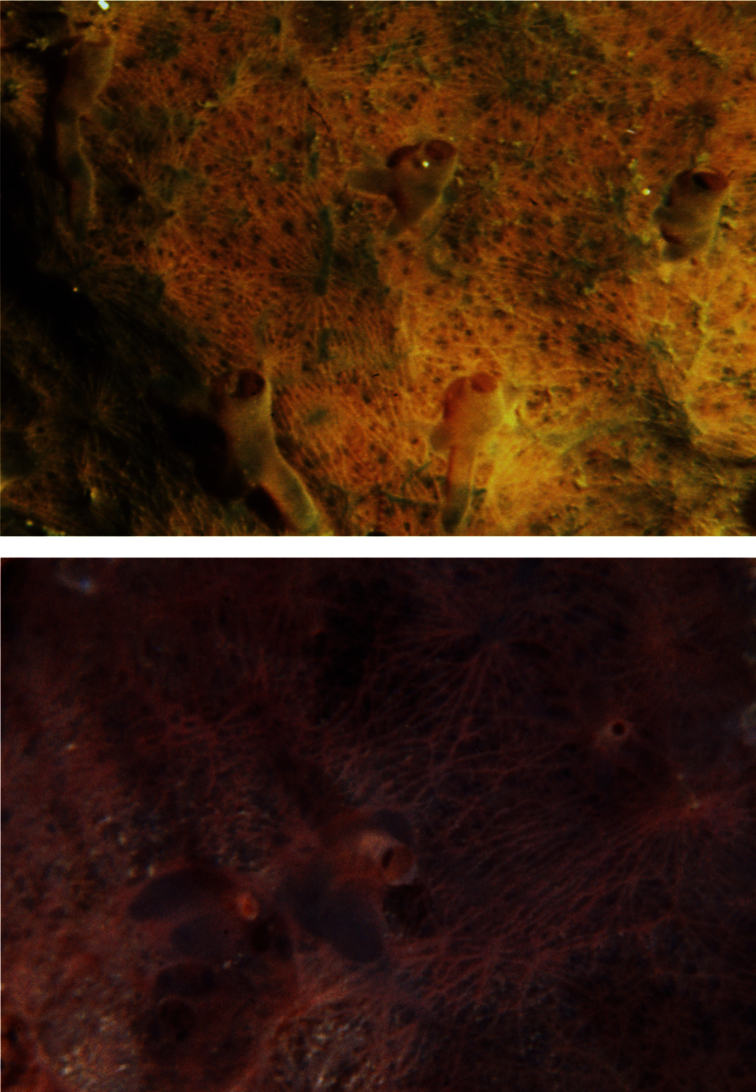
*Hexadella* spp. Underwater photographs of two specimens with the surface ornamentation and oscular funnels typical of the genus. Colour *in vivo* is not diagnostic at the species level.

#### Hexadella pruvoti

Topsent, 1896

http://species-id.net/wiki/Hexadella_pruvoti

Fig. 39

Hexadella pruvoti Topsent, 1896: 120 (*partim*).

##### Description.

Growth form thinly encrusting and lobate, in large patches. Colour bright yellow *in vivo*, dark purple in alcohol after releasing a yellowish fluid. Surface finely conulose, entirely wrinkled by small evident collagenous reinforcements irregularly crossing and converging towards small conules, with inconspicuous inhalant apertures surrounding the tiny conules armed by debris. Large oscules *in vivo*, not visible after fixation in ethanol. Ectosome with bundles of collagen fibrils. Choanosome fragile with large clusters of spherulous cells with large inclusions of heterogeneous size, containing microgranules and microgranular cells. Choanocyte chambers (ca. 40 × 20 µm in diameter) eurypylous, densely packed with 40–60 choanocytes. Bacteria in the mesohyl. Aerophobins 1 and 2 compounds with medium-high natural toxicity.

##### Habitat.

Cave, rocky cliffs. Bathymetric range 10–35 m.

##### Mediterranean caves.

Blava, Blue, Misidacis caves (Balearic Sea); Corail Cave (Gulf of Lions); Trypia Spilia Cave (Aegean Sea) (Martì et al. 2004; Turon et al. 2009; Reveillaud et al. 2010, 2012; Pronzato and Manconi 2011; Gerovasileiou and Voultsiadou 2012).

#### Hexadella racovitzai

Topsent, 1896

http://species-id.net/wiki/Hexadella_racovitzai

Fig. 39

Hexadella racovitzai Topsent, 1896: 119 (*partim*).

##### Description.

Growth form encrusting, thin, with lobes in large patches. Colour faded to pale pink *in vivo*, brownish in ethanol after releasing of a yellow fluid. Surface highly wrinkled by small evident collagenous reinforcements irregularly crossing and converging towards small conules; well developed (when compared to *Hexadella pruvoti* and *Hexadella crypta*) star-shaped network of subdermal canals converging towards oscula; inhalant apertures inconspicuous. Oscules wide, at the apices of short chimneys. Ectosome notably thick. Choanosome soft, fleshy and fragile, difficult to cut. Large clusters of spherulous cells, common at the body surface, with large inclusions containing microgranules and microgranular cells; choanocyte chambers eurypylous (30±6.3 × 19±2 µm on average) in dense clusters. High natural toxicity.

##### Habitat.

Cave, coralligenous community, rocky cliffs. Bathymetric range 25–38 m. Already deeper than 100 m.

##### Mediterranean caves.

La Catedral Cave (Balearic Sea); Corail Cave (Gulf of Lions); Leuca caves (Ionian Sea); Stražica Cave (Northern Adriatic Sea); Farà, Agios Vasilios, Alikes caves (Aegean Sea) (Pulitzer-Finali and Pronzato 1980; Bibiloni et al. 1989; Novosel et al. 2002; Reveillaud et al. 2010; Pronzato and Manconi 2011; Gerovasileiou and Voultsiadou 2012).

#### Hexadella topsenti

Reveillaud, Allewaert, Pérez, Vacelet, Banaigs and Vanreusel, 2012

http://species-id.net/wiki/Hexadella_topsenti

Fig. 39

Hexadella topsenti Reveillaud, Allewaert, Pérez, Vacelet, Banaigs and Vanreusel, 2012: 242.

##### Description.

Growth form encrusting, lobate and thin. Colour bright to dark pink, to purple *in vivo* (brighter and deeper pink than *Hexadella racovitzai*), changing to brownish after releasing of a yellow fluid in ethanol. Surface smooth with subdermal canals, and wrinkled by small evident collagenous reinforcements irregularly crossing and converging towards small, tiny conules; foreign inclusions present. Inhalant apertures inconspicuous; oscules small, chimney-like, abundant, scattered. Ectosome with some bundles of collagen fibrils and a developed lacunar system. Spherulous cells in large clusters with large inclusions containing microgranules and microgranular cells. Choanocyte chambers (35 × 20 µm in diameter), choanocytes larger than in *Hexadella racovitzai*. Rod-shaped bacteria in the mesohyl. Low-moderate natural toxicity.

##### Habitat.

Coralligenous cliff, cave.

##### Mediterranean caves.

Corail Cave (Gulf of Lions) (Reveillaud et al. 2012).

##### Remarks.

See the original description for more details and figures (Reveillaud et al. 2012).

## Conclusive remarks

Mediterranean marine caves host one of the least investigated biocoenosis. Despite the difficulties of accessing these biotopes, their horny sponge fauna was recorded in 51 papers published between 1958 and 2012, that focused on marine submerged and semi-submerged caves, mostly along the Italian coasts (Fig. 1; Table 1). Several papers refer each to a single or very few sponge records. Caves of the Levant Basin and the northern African coasts are scarcely or absolutely not investigated. Moreover, each paper reports a species list which is spot data series with no replicas to indicate the real taxonomic richness and/or population dynamics.

The present faunistic assessment, based on literature and new data, results in high values of taxonomic richness of Mediterranean cave-dwelling horny sponges with 4 orders, 9 families, 19 genera and 40 species (Table 2) recorded in 105 out of *ca*. 150 investigated caves. The new data refer to the first record of 18 species in recently investigated karstic caves (Fig. 1; Tables 1, 2) namely, 14 species from the Capo Caccia-Isola Piana MPA (Galatea, Falco, Bisbe caves), six species from the Plemmirio MPA (Mazzere, Gamberi, Gymnasium caves), and nine species from the Pelagie MPA (Taccio Vecchio I Cave, Lampedusa) (Manconi et al. 2011; Cadeddu 2012). The present synthesis demonstrates how cave-dwelling horny sponges are representatives of the taxon Porifera in the whole Mediterranean basin thus confirming the high affinity of this pool of species for marine caves; indeed 70% of Mediterranean species (40 out of 57) were recorded to date in marine caves. Species endemic to the Mediterranean Sea harboured in marine caves number 14 with an endemicity value of 35%.

A few species such as *Coscinoderma sporadense*, *Euryspongia raouchensis*, *Hexadella crypta* and *Hexadella topsenti* are, however, recorded only once, exclusively from their type locality. Although some few species are reported only from caves, the present overview cannot assert the existence of horny sponge species exclusively restricted to cave habitats. The topographic distribution of horny sponges in each investigated cave is restricted to the cave entrance until the semi-dark zone, while no record is reported for confined zones of the caves matching those reported by Pouliquen (1972).

The census of marine caves sponge fauna is characterized by non-homogeneity of sampling methods and efforts, limiting the possibilities of exhaustive comparative analysis of this biocoenosis in the whole of the Mediterranean Sea. Results highlight also that Mediterranean marine caves host seven horny sponges species listed in the appendices II and III of the Barcelona Convention as “protected species of the protocol SPA/BIO”, namely *Aplysina aerophoba*, *Aplysina cavernicola*, *Sarcotragus foetidus*, *Sarcotragus pipetta*, *Spongia lamella*, *Spongia officinalis* and *Spongia zimocca*. They belong to protected biocoenosis of marine caves registered as Habitat II.4.3, Habitat IV.3.2, and Habitat V.3.2 matching the category of mid-littoral caves, semi-dark caves, and dark caves (Relini and Giaccone 2009; Relini and Tunesi 2009). These horny sponge species have a high economic value and are reported as endangered (see Pronzato et al. 2003). The entire data set highlights how marine caves represent a hotspot of biodiversity needing further scientific investigation and appropriate conservation measures that can exert a key role in supporting survival and random genetic reassortment of populations belonging to these species (*i.e*. caves as reserves of genetic biodiversity) in all Mediterranean biotopes. This matches perfectly both the UE Habitat 8330 strategy of conservation and the biodiversity assessment of Mediterranean species at risk in the progressive environmental/climatic change of the entire basin.

## Supplementary Material

XML Treatment for Aplysilla rosea

XML Treatment for Chelonaplysilla noevus

XML Treatment for Darwinella simplex

XML Treatment for Spongionella gracilis

XML Treatment for Spongionella pulchella

XML Treatment for Dysidea avara

XML Treatment for Dysidea fragilis

XML Treatment for Dysidea incrustans

XML Treatment for Dysidea tupha

XML Treatment for Euryspongia raouchensis

XML Treatment for Pleraplysilla minchini

XML Treatment for Pleraplysilla spinifera

XML Treatment for Ircinia dendroides

XML Treatment for Ircinia oros

XML Treatment for Ircinia paucifilamentosa

XML Treatment for Ircinia retidermata

XML Treatment for Ircinia variabilis

XML Treatment for Sarcotragus fasciculatus

XML Treatment for Sarcotragus foetidus

XML Treatment for Sarcotragus pipetta

XML Treatment for Sarcotragus spinosulus

XML Treatment for Coscinoderma sporadense

XML Treatment for Hippospongia communis

XML Treatment for Spongia lamella

XML Treatment for Spongia nitens

XML Treatment for Spongia officinalis

XML Treatment for Spongia virgultosa

XML Treatment for Spongia zimocca

XML Treatment for Cacospongia mollior

XML Treatment for Cacospongia proficens

XML Treatment for Cacospongia scalaris

XML Treatment for Fasciospongia cavernosa

XML Treatment for Hyrtios collectrix

XML Treatment for Halisarca dujardini

XML Treatment for Aplysina aerophoba

XML Treatment for Aplysina cavernicola

XML Treatment for Hexadella crypta

XML Treatment for Hexadella pruvoti

XML Treatment for Hexadella racovitzai

XML Treatment for Hexadella topsenti
